# DSR-VGG: A Lightweight Depthwise Separable Residual VGG Architecture for Nasopharyngeal Histopathological Image Classification

**DOI:** 10.3390/diagnostics16203406

**Published:** 2026-10-20

**Authors:** Muhammad Kabir Abdullahi, Sarina Mansor, Mohammad Faizal Ahmad Fauzi

**Affiliations:** 1 Faculty of Artificial Intelligence and Engineering, Multimedia University (MMU), Cyberjaya 63100, Malaysia; abdullahimkay@gmail.com; 2 School of Digital Health, KPJ Healthcare University, Nilai 71800, Malaysia; faizal.fauzi@kpju.edu.my

**Keywords:** nasopharyngeal carcinoma (NPC), histopathological image classification, deep learning, convolutional neural networks (CNNs), depthwise separable convolution, residual learning, lightweight neural networks, digital pathology, medical image analysis, computer-aided diagnosis (CAD)

## Abstract

**Background/Objectives:** Deep convolutional neural networks achieve strong histopathological image-classification performance but often require substantial computational resources. This study proposes DSR-VGG, a lightweight Depthwise Separable Residual VGG architecture for nasopharyngeal histopathological image classification. **Methods:** DSR-VGG comprises six Depthwise Separable Blocks with residual projection shortcuts, global average pooling, dropout regularization, and a classification layer. An inverse-frequency weighted random sampling strategy was used to address severe class imbalance. The primary evaluation used 88,002 nasopharyngeal histopathology patches from four classes: Normal, lymphoid hyperplasia, nasopharyngeal inflammation, and nasopharyngeal carcinoma. Training was repeated using three random seeds, with supplementary public datasets used to assess cross-dataset architectural robustness. **Results:** Across three runs, DSR-VGG achieved 99.764 ± 0.049% accuracy, 99.070 ± 0.365% balanced accuracy, and 99.219 ± 0.215% macro-F1. The model contained only 0.528 million parameters, required approximately 0.31 GFLOPs, and achieved 2.38 ms single-image inference latency under the stated GPU benchmarking conditions. Although several baseline architectures achieved marginally higher accuracy, DSR-VGG maintained competitive predictive performance with substantially lower computational complexity. **Conclusions:** DSR-VGG provides a favorable accuracy–complexity trade-off for histopathological patch classification. However, patient-/WSI-disjoint evaluation, independent multi-center validation, and whole-slide assessment remain necessary before clinical deployment.

## 1. Introduction

Nasopharyngeal carcinoma (NPC) is a malignant epithelial tumor originating from the nasopharyngeal mucosa and represents one of the most prevalent head-and-neck cancers in Southeast Asia, Southern China, and North Africa [1,2,3,4,5]. Due to its deep anatomical location and nonspecific early-stage symptoms, NPC is frequently diagnosed at advanced stages, leading to poor prognosis and increased treatment complexity. Early and accurate diagnosis is therefore critical to improving patient outcomes and survival rates. Histopathological examination of nasopharyngeal biopsy specimens remains the gold standard for NPC diagnosis, providing definitive evidence of malignant cellular and tissue-level abnormalities [6,7]. Beyond histopathological diagnosis, multimodal imaging also contributes to the broader characterization and management of NPC, including assessment of treatment response. Recent evidence has highlighted the potential value of [18F] FDG PET/CT-derived parameters for predicting response to neoadjuvant chemoradiotherapy in patients with NPC [8].

Recent advances in whole-slide imaging (WSI) technology have enabled the digitization of histopathological slides, facilitating the development of computer-aided diagnostic systems [9]. Digital pathology has emerged as a promising approach for improving diagnostic efficiency, reducing inter-observer variability, and assisting pathologists in large-scale screening applications. However, manual examination of histopathological images remains labor-intensive, time-consuming, and highly dependent on specialist expertise [10]. Furthermore, the increasing volume of pathology data generated in modern healthcare systems has created a growing demand for automated and intelligent diagnostic tools capable of supporting clinical decision-making [11].

The rapid development of deep learning, particularly Convolutional Neural Networks (CNNs), has revolutionized medical image analysis by enabling automatic extraction of hierarchical image features directly from raw image data. Unlike conventional machine learning methods that rely heavily on handcrafted features, CNNs learn discriminative representations through end-to-end optimization, allowing them to achieve remarkable performance across various image classification tasks [12,13]. In digital pathology, CNN-based models have demonstrated significant success in detecting and classifying multiple cancer types, including breast, lung, colorectal, and head-and-neck cancers. These systems have consistently outperformed traditional feature-engineering approaches by capturing complex tissue morphology, cellular organization, and pathological patterns [12,13,14].

Despite these achievements, histopathological image classification remains a challenging problem. Histopathological datasets often exhibit substantial intra-class variability, inter-class similarity, staining inconsistencies, imaging artifacts, and scanner-dependent variations [15]. In addition, datasets frequently suffer from severe class imbalance, where malignant and benign categories are represented by significantly different numbers of samples [16]. These factors can negatively affect model generalization and increase susceptibility to overfitting [17]. Such challenges are particularly evident in nasopharyngeal biopsy image datasets, where subtle tissue-level differences must be distinguished across multiple pathological categories.

To address these challenges, researchers have explored increasingly sophisticated deep neural network architectures. VGGNet [18] introduced a simple yet powerful framework based on sequential convolutional layers, providing strong feature-extraction capability and serving as the foundation for numerous medical imaging applications. Subsequently, ResNet [19] introduced residual learning mechanisms that alleviate gradient degradation and facilitate the training of deeper networks through shortcut connections. More recent architectures, including DenseNet [20], EfficientNet [21], ConvNeXt [22], and Vision Transformers [23], have further improved classification accuracy through enhanced feature reuse and advanced architectural designs.

Although these architectures achieve excellent predictive performance, they typically require millions of trainable parameters and substantial computational resources. Such requirements can limit practical deployment in clinical environments, particularly in resource-constrained settings where computational efficiency, memory consumption, and inference speed are important considerations. Consequently, there is growing interest in developing lightweight deep learning architectures that can maintain competitive classification performance while reducing computational complexity.

One of the most successful approaches for constructing efficient CNNs [21,24,25] is the use of Depthwise Separable Convolutions (DSCs). Unlike standard convolutions, which simultaneously learn spatial and channel relationships, DSCs decompose the operation into separate depthwise and pointwise stages. This factorization significantly reduces the number of parameters and floating-point operations (FLOPs) while preserving feature-extraction capability. Architectures such as MobileNet [24] and Xception [25] have demonstrated the effectiveness of DSCs for efficient visual recognition tasks. Nevertheless, the direct application of lightweight architectures to histopathological image classification may not always achieve optimal performance due to the complex and fine-grained nature of pathological tissue patterns [26].

Residual learning provides a complementary solution by improving information propagation throughout the network and facilitating the training of deeper models. Residual shortcut connections enable feature reuse, mitigate gradient degradation, and improve convergence stability [19,20]. Combining residual learning with depthwise separable convolutions therefore offers the potential to construct compact architectures that simultaneously achieve computational efficiency and strong representational capability [27].

Motivated by these observations, this study proposes an Efficient Depthwise Separable Residual VGG Architecture for nasopharyngeal biopsy image classification. The proposed architecture integrates lightweight Depthwise Separable Blocks (DSBlocks) with linear projection residual shortcuts within a VGG-inspired framework. By replacing computationally expensive standard convolutions with depthwise separable operations and introducing residual feature-propagation mechanisms, the proposed model significantly reduces parameter count while preserving discriminative feature-extraction capability.

Furthermore, an inverse-frequency weighted random sampling strategy is employed during training to address severe class imbalance in the NPC dataset, thereby increasing the sampling probability of underrepresented classes.

The proposed framework is primarily evaluated using a large-scale multi-center nasopharyngeal histopathological dataset comprising 88,002 image patches distributed across four diagnostic categories: Nasopharyngeal Carcinoma (NPC), Nasopharyngeal Inflammation (NPI), Lymphoid Hyperplasia (LHP), and Normal tissue. This private NPC dataset forms the central clinical basis of the study; therefore, all primary diagnostic-performance claims, class-imbalance analysis, ablation experiments, and comparisons with baseline architectures are based on this dataset.

To further examine whether the proposed lightweight architecture can maintain effective performance under different data distributions, supplementary experiments are conducted using several publicly available medical image-classification benchmarks. These datasets are included to assess cross-dataset robustness and computational efficiency rather than to provide external clinical validation for NPC diagnosis, because they differ from the primary dataset in disease site, class definitions, staining protocol, and, in one case, imaging modality. The results from these supplementary datasets should therefore be interpreted as evidence of architectural robustness across diverse medical-image classification settings.

The primary contributions of this work are summarized as follows:

1. A novel lightweight residual VGG architecture is proposed for efficient nasopharyngeal biopsy image classification.
2. Custom Depthwise Separable Blocks (DSBlocks) are introduced to substantially reduce computational complexity and parameter count while maintaining strong feature-extraction capability.
3. A linear projection residual mapping mechanism is incorporated to enhance information flow, improve optimization stability, and facilitate deeper feature learning.

The remainder of this paper is organized as follows. Section 2 reviews related studies in histopathological image classification, lightweight deep learning architectures, depthwise separable convolutions, and residual learning. Section 3 describes the dataset, preprocessing pipeline, and proposed DSR-VGG architecture. Section 4 presents the experimental results and comparative analysis. Finally, Section 5 concludes the paper and discusses future research directions.

## 2. Related Works

### 2.1. Deep Learning for Histopathological Image Classification

Histopathological image analysis has become one of the most active research areas in computer-aided diagnosis due to the increasing availability of whole-slide imaging (WSI) systems and advances in deep learning [8,9,10,11]. Traditional machine learning approaches relied on handcrafted texture, color, and morphological descriptors combined with classifiers such as Support Vector Machines (SVMs) and Random Forests. However, these methods often struggled to capture the complex tissue structures and cellular patterns present in pathological images [28,29,30].

The emergence of deep convolutional neural networks (CNNs) significantly improved feature extraction and classification performance by learning hierarchical representations directly from image data. CNN-based approaches have demonstrated remarkable success in various cancer diagnosis tasks, including breast cancer [31], colorectal cancer [32], lung cancer [33], and head-and-neck cancer histopathology [34]. By automatically learning discriminative tissue features, deep learning models reduce the dependency on handcrafted feature engineering while improving robustness and generalization. Abdulaal et al. [35] introduced a hierarchical self-learning algorithm that integrates transfer learning with the Inception-V3 architecture to effectively correct noisy labels in breast cancer sub-images, ultimately reaching an accuracy of 99.1%. Similarly addressing diagnostic limitations, Ukwuoma et al. [36] proposed a novel spatiochannel attention network designed to blend both low-level and high-level features for early cancer detection, while incorporating Explainable AI (XAI) tools like LIME and SHAP to provide clinical interpretability for its high-accuracy predictions. Complementing these advancements in feature extraction and transparency, Balasubramanian et al. [37] utilized an ensemble deep learning approach featuring VGG16 and ResNet50 architectures alongside a specialized image patching technique to manage high-resolution Whole Slide Images (WSIs), achieving a superior validation accuracy of 98.43% on the BreakHis dataset. Together, these research efforts demonstrate a critical evolution in computer-aided diagnosis by prioritizing noisy data management, explainable decision-making, and robust ensemble methodologies to enhance the reliability and clinical trust of automated histopathological analysis.

Despite these advances, histopathological image classification remains challenging due to variations in staining procedures, imaging devices, tissue heterogeneity, and severe class imbalance [38]. These challenges are particularly evident in nasopharyngeal carcinoma (NPC) datasets, where malignant samples often dominate the dataset distribution. Consequently, there is a continuing need for efficient deep learning architectures capable of extracting robust pathological features while maintaining computational efficiency.

Beyond conventional handcrafted feature engineering and end-to-end deep learning, histomics provides a complementary framework for quantitative tissue phenotyping in computational pathology. Histomic representations explicitly characterize biologically meaningful properties of histological tissue, including cellular morphology, spatial organization, tissue architecture, and multi-scale structural patterns. Neha et al. [39] described histomics as an intermediate phenotype representation between raw histological images and downstream clinical inference, providing structured and interpretable descriptors that complement the latent feature representations learned by end-to-end CNNs. While CNN-based approaches such as the present DSR-VGG model emphasize automatic discriminative feature learning and efficient classification, histomics can provide greater morphological interpretability and facilitate analysis of how tissue organization contributes to prediction. These paradigms are therefore complementary, and future computational pathology systems may benefit from integrating efficient deep representations with explicit morphology- and phenotype-based descriptors.

### 2.2. CNN Architectures for Medical Image Analysis

The success of deep learning in medical imaging has largely been driven by advances in CNN architectures. Early models such as AlexNet [40] demonstrated the effectiveness of deep convolutional feature extraction, while VGGNet introduced a simple yet powerful architecture based on stacked 3 × 3 convolutional layers. VGG networks became widely adopted in medical image analysis due to their straightforward design and strong feature-learning capability. Subsequent architectures focused on improving optimization and representation learning. ResNet introduced residual learning through skip connections, enabling the successful training of substantially deeper networks and mitigating the vanishing gradient problem. DenseNet further enhanced information flow by establishing dense connections between layers, improving feature reuse and gradient propagation.

Güler et al. [41] introduced a hybrid diagnostic framework for brain tumors that integrates CNN-based feature extraction with optimized machine learning classifiers, achieving a definitive 100% accuracy through grid-search parameter optimization and ResNet architecture refinement. Addressing the need for comprehensive diagnostic views, Tang et al. [42] proposed a multiscale adaptive Transformer (MATR) designed for SPECT and MRI image fusion, which employs adaptive convolutions and long-range dependency modeling to simultaneously preserve metabolic information and high-resolution anatomical details. In a comparative analysis of model suitability across different pathologies, Dai et al. [43] demonstrated that while DenseNet201 provided superior performance for COVID-19 detection on smaller datasets with a 91.1% accuracy, ResNet-50 proved more effective for large-scale breast cancer datasets, reaching an accuracy of 89.7%. Complementing these findings, Surendren et al. [44] evaluated various deep learning architectures for skin cancer classification, concluding that DenseNet’s ability to promote feature reuse and propagation resulted in a peak accuracy of 84.35% for distinguishing between benign and malignant lesions compared to VGG and ResNet models.

More recently, EfficientNet proposed a compound scaling strategy that jointly scales network depth, width, and input resolution, achieving superior accuracy–efficiency trade-offs compared to conventional CNN architectures. Shashidhar et al. [45] developed a brain tumor classification framework using the EfficientNet (EffNet) architecture, which achieved an accuracy of 98.08% in identifying glioma, pituitary, and meningioma tumors from MRI scans. Similarly, Das et al. [46] utilized EfficientNet-B0 for multi-class skin lesion classification, reaching a peak accuracy of 97.15% by integrating test-time augmentation (TTA) and Monte Carlo dropout to quantify predictive uncertainty. Both studies highlight that EfficientNet’s compound scaling method allows for high diagnostic precision while remaining computationally efficient, making these models ideal for real-time clinical deployment. Although these models achieve excellent performance in histopathological image classification, many require substantial computational resources and memory, limiting their applicability in resource-constrained environments.

### 2.3. Lightweight Deep Learning Architectures

The growing demand for deployment on embedded devices and edge computing platforms has motivated the development of lightweight CNN architectures. These networks aim to reduce computational complexity while maintaining competitive classification accuracy.ShuffleNet introduced grouped convolutions and channel shuffling operations to decrease computational cost. MobileNet employed depthwise separable convolutions to drastically reduce the number of parameters and floating-point operations while preserving classification performance. MobileNet subsequently became one of the most influential lightweight architectures for mobile and embedded vision systems. EfficientNet further improved efficiency through neural architecture search and compound model scaling.

Al-Milaji et al. [47] proposed a lightweight deep learning model optimization (LDLMO) framework consisting of just five convolutional layers, which employs the particle swarm optimization (PSO) algorithm to find the ideal number of kernel filters for medical image classification. Their methodology incorporates color deconvolution for stain separation and a second-pass training phase to exclude uncertain data, resulting in high-performance generalization across various datasets such as MedMNIST and brain tumor MRI. Building on the theme of efficient architectures, Das et al. [44] developed a robust multi-class skin lesion classification system using the EfficientNet-B0 model trained on the HAM10000 dataset. Through the integration of transfer learning, full-network fine-tuning, and inference enhancements like test-time augmentation (TTA) and Monte Carlo (MC) dropout, they achieved a peak classification accuracy of 97.15%, demonstrating the model’s potential as a clinical decision support tool. Furthering the advancement of lightweight diagnostic tools, Zhou et al. [48] introduced a spiking cortical model-based global and local (SCM-GL) attention module that decomposes feature maps into parallel local and global components to improve feature extraction in architectures like MobileNetV3 and ShuffleNetV2. By simulating human visual perception to highlight suspected lesions while suppressing background noise, this mechanism significantly improved classification performance across brain MR, chest X-ray, and osteosarcoma datasets, outperforming state-of-the-art attention modules in both accuracy and F1 scores.

Although lightweight architectures significantly reduce computational requirements, many experience reduced representational capacity when applied to complex histopathological datasets. Consequently, balancing computational efficiency and discriminative feature learning remains an important research challenge.

### 2.4. Depthwise Separable Convolutions

Depthwise separable convolution is a factorized convolution operation that decomposes a standard convolution into two sequential stages: depthwise convolution and pointwise convolution. Instead of simultaneously learning spatial and channel correlations, the operation separates these processes into independent spatial filtering and channel projection stages.

Zhou et al. [49] proposed the Multi-Attention and Depthwise Separable Convolution U-Net (MDSU-Net), which integrates dual attention and attention gates to capture rich contextual details while utilizing depthwise separable convolution (DSC) to reduce model complexity without sacrificing segmentation efficacy across diverse medical datasets. Expanding on the use of efficient architectures for diagnostic tasks, Huang et al. [50] developed the Dense Depthwise Separable Network (DDSNet), a lightweight model that combines dense connections with DSC to achieve tumor classification results that are 32x smaller and 5x faster than standard DenseNet models, facilitating real-world deployment on IoT devices. Furthering the application of DSC in complex imaging, Dang et al. [27] introduced a lightweight classification framework for hyperspectral images (HSI) that stacks residual units with fewer parameters to extract spatial-spectral features, achieving high classification accuracies up to 99.96% while significantly lowering computational costs compared to traditional 3D-CNN approaches.

Several studies have further extended depthwise separable convolution through multi-scale learning, pyramid convolution structures, and hybrid residual frameworks. These investigations demonstrate that depthwise separable convolutions can effectively preserve feature representation while substantially reducing computational overhead, making them highly suitable for large-scale image classification tasks.

### 2.5. Residual Learning and Skip Connections

As CNN depth increases, optimization becomes increasingly difficult due to gradient degradation and information loss. Residual learning was introduced to address this issue by incorporating shortcut connections that allow information to bypass intermediate layers. The residual framework enables the network to learn residual mappings rather than direct transformations, facilitating gradient propagation during backpropagation and improving convergence stability. Residual connections have been widely adopted across numerous state-of-the-art architectures because they improve feature preservation and support deeper network construction.

Shashidhar et al. [45] proposed a robust brain tumor classification framework using the EfficientNet (EffNet) architecture, which employs a unique compound scaling method to balance network depth, width, and resolution, ultimately achieving a classification accuracy of 98.08% across four distinct classes. Similarly targeting diagnostic precision, Zahoor et al. [51] introduced Res-BRNet, a novel architecture that integrates spatial and residual blocks with regional and boundary-based operations to capture complex tumor patterns and textural variations, reaching an accuracy of 98.22% and significantly outperforming standard CNN models. Addressing the limitations of shallow networks, Ashwini B. et al. [50] developed the ESRNet (Efficient Skip Connections-based Residual Network), which utilizes identity mapping through skip connections to mitigate the vanishing gradient problem and facilitate in-depth feature learning, resulting in an exceptional median accuracy of 99.62%. Providing a theoretical foundation for these advancements, Xu et al. [52] conducted a comprehensive survey on skip connections, concluding that they are essential for optimizing deep neural networks through residual learning, thereby enhancing accuracy and stability in complex medical image analysis tasks. Together, these research efforts highlight a critical evolution in neuro-oncology diagnostics by prioritizing architectural depth, optimized gradient flow, and multi-scale feature extraction to ensure reliable and efficient automated classification.

Projection shortcuts further extend residual learning by aligning feature dimensions between the main processing path and the shortcut branch. Such mechanisms are particularly beneficial when spatial resolution changes occur due to pooling or strided convolution operations. In medical image analysis, residual learning has consistently demonstrated improvements in classification accuracy and training stability.

### 2.6. Hybrid Lightweight Residual Networks

Recent research has increasingly focused on combining lightweight convolutional operations with residual learning mechanisms. MobileNetV2 introduced inverted residual bottlenecks that integrate depthwise separable convolutions with residual shortcuts. Similarly, EfficientNet, MixNet, and various lightweight residual architectures exploit efficient convolutional operators while maintaining strong feature-extraction capability.

Kaur et al. [53] proposed the Efficient Skip Connections-based Residual Network (ESRNet) for brain tumor classification, demonstrating the effectiveness of efficient skip connections for supporting information flow and feature reuse in medical image classification. Asif et al. [54] proposed DCDS-Net, a deep transfer network for gastrointestinal disease diagnosis that integrates depth-wise separable convolutions with residual connections and densely connected blocks, achieving a high classification accuracy of 99.33% through a specialized block-wise fine-tuning strategy. Similarly, Sarkar et al. [55] introduced a deep depthwise separable residual convolutional network for melanoma classification that leverages separable convolutions to significantly reduce parameter complexity and execution time while utilizing residual learning to reach a state-of-the-art accuracy of 99.50%. Yu et al. [56] developed MedLiteNet, a lightweight CNN-Transformer hybrid for skin-lesion segmentation that employs depth-wise Mobile Inverted Bottleneck blocks and boundary-aware self-attention to capture global context without the high computational costs typical of standard Transformers. Complementing these approaches, Tummala et al. [57] presented the ResMHCNN architecture, which utilizes residual multi-head convolution with channel-wise attention to learn importance weights for different kernel sizes, enabling accurate classification across multiple cancer types with parameter budgets as low as 0.226 million. Together, these studies demonstrate a critical evolution in medical imaging by prioritizing parameter-efficient architectures, robust gradient flow, and hybrid feature extraction to ensure reliable clinical performance in resource-constrained environments.

Hybrid architectures generally provide superior parameter efficiency compared with conventional CNNs while preserving competitive classification performance. However, many existing approaches remain optimized for natural image datasets and may not fully exploit the unique structural characteristics of histopathological images. Furthermore, several architectures continue to rely on relatively large parameter budgets, limiting deployment in resource-constrained environments.

### 2.7. Research Gap

Although significant progress has been achieved in histopathological image classification, several limitations remain. First, many high-performing CNN architectures rely on millions of trainable parameters and substantial computational resources. Second, lightweight networks often experience reduced representational power when handling highly heterogeneous pathological tissue patterns. Third, limited studies have investigated the integration of VGG-style hierarchical feature extraction, depthwise separable convolutions, and residual projection shortcuts specifically for nasopharyngeal histopathological image classification.

To address these limitations, this work proposes DSR-VGG, a lightweight residual VGG architecture built upon custom Depthwise Separable Blocks (DSBlocks) and linear projection residual mappings. The proposed design aims to preserve discriminative feature-extraction capability while significantly reducing parameter count and computational complexity, making it suitable for efficient and accurate nasopharyngeal biopsy image classification.

## 3. Materials and Methodology

The proposed framework introduces an optimized deep learning workflow designed to address class imbalance and high computational overhead in large-scale image classification tasks. The methodology is split into three primary phases: data preprocessing and weighted random sampling, the architecture of the proposed hybrid Depthwise Separable Residual VGG network, and the optimization and training configuration.

### 3.1. Experimental Datasets and Study Scope

This study uses one primary private dataset and several supplementary public benchmark datasets. The central objective is to develop and evaluate DSR-VGG for multi-class nasopharyngeal histopathological image classification. Accordingly, the private nasopharyngeal carcinoma (NPC) dataset is designated as the primary clinical dataset and serves as the basis for the principal evaluation of diagnostic performance, class-imbalance mitigation, ablation analysis, and comparison with competing deep learning architectures.

The supplementary public datasets, comprising HER2 histopathology, NCT-CRC-HE-100K, HistoSet-5×14, and CT Kidney images, are used solely as cross-dataset robustness benchmarks. These datasets contain different organs, diagnostic tasks, class structures, staining protocols, and image-acquisition characteristics. In particular, the CT Kidney dataset represents a radiological imaging modality rather than histopathology. Therefore, these experiments are not considered external clinical validation of the NPC classifier. Instead, they evaluate whether the proposed architecture retains a favorable accuracy-efficiency trade-off across diverse medical image-classification settings.

An overview of the primary NPC dataset and the supplementary public benchmark datasets is provided in Table 1. The table summarizes the modality, classification task, number of classes, dataset size, experimental role, and data partition for each dataset.

As shown in Table 1, the NPC private dataset is the primary dataset used to assess the clinical classification performance of DSR-VGG. The remaining datasets are included as supplementary benchmarks to evaluate the robustness and computational efficiency of the proposed architecture across different medical image-classification tasks.

The following subsections describe the primary NPC dataset and the supplementary public datasets used in this study.

#### 3.1.1. NPC Private Dataset

**Figure 1:**
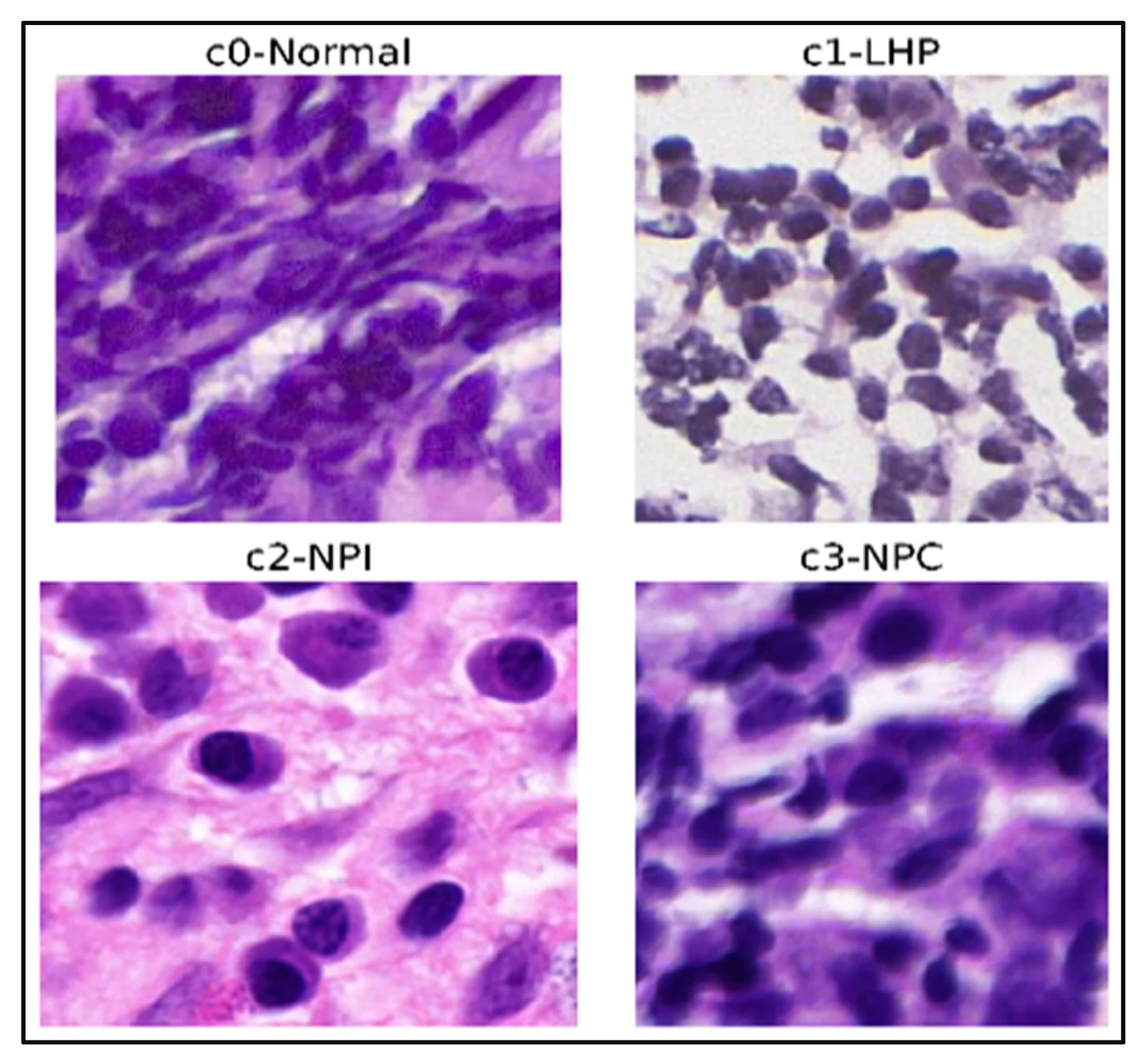
Representative samples from the NPC private histopathology dataset. The source WSIs from Sarawak General Hospital (SGH) were digitized at 20× magnification, whereas those from Hospital Kuala Lumpur (HKL) were digitized at 40× magnification.

The private dataset is total of 88,002 histopathological patch samples extracted from Whole Slide Images (WSIs). The cohort is categorized into four distinct clinical classes: Normal tissue, Lymphoid Hyperplasia (LHP), Nasopharyngeal Inflammation (NPI), and Nasopharyngeal Carcinoma (NPC) as shown in Figure 1. The underlying data was collected through a multi-center collaboration involving the Sarawak General Hospital (SGH) and Hospital Kuala Lumpur (HKL). The NPC dataset was annotated by three expert pathologists using the web-based Cytomine 3.10 platform. The available dataset documentation does not specify whether each image was independently reviewed by all three pathologists or whether a formal consensus/adjudication procedure was applied. Furthermore, no formal interobserver-agreement statistic, such as Cohen’s or Fleiss’ kappa, was available for the present study.

The dataset was partitioned into static subsets consisting of 44,002 training samples (50%), 22,001 validation samples (25%), and 21,999 testing samples (25%). During the original dataset preparation procedure, the WSIs were first tiled into image patches, after which the resulting patches were randomly assigned to the training, validation, and test subsets. The partitioning was therefore performed at the patch level rather than at the patient or WSI level. Consequently, patches originating from the same WSI may occur in more than one subset, and the reported results should therefore be interpreted as internal patch-level classification performance rather than patient-level or WSI-level diagnostic performance. The explicit class-wise numerical distribution across these splits is detailed in Table 2.

A further challenge of the dataset is variation in image acquisition conditions between the two contributing institutions. WSIs obtained from SGH were digitized at 20× magnification, with a typical resolution of approximately 125,000 × 295,000 pixels, whereas WSIs from HKL were digitized at 40× magnification, with a typical resolution of approximately 250,000 × 572,000 pixels. These differences in magnification, spatial resolution, and image acquisition introduce additional heterogeneity into the dataset and may influence the scale and appearance of histopathological structures presented to the model.

The difference in acquisition magnification also implies a difference in the physical tissue area represented by a fixed number of pixels. During the original dataset preparation, the higher-resolution 40× HKL images were downsampled by approximately a factor of two to reduce the scale discrepancy relative to the 20× SGH images before the final model-input standardization. The resulting patches from both sources were subsequently resized to 224 × 224 pixels for network input. This preprocessing reduced the gross magnification difference between the two acquisition settings; however, it should not be interpreted as exact physical-scale calibration because an explicitly fixed microns-per-pixel value was not applied using scanner metadata.

#### 3.1.2. Public Datasets

(i) HER2

**Figure 2:**
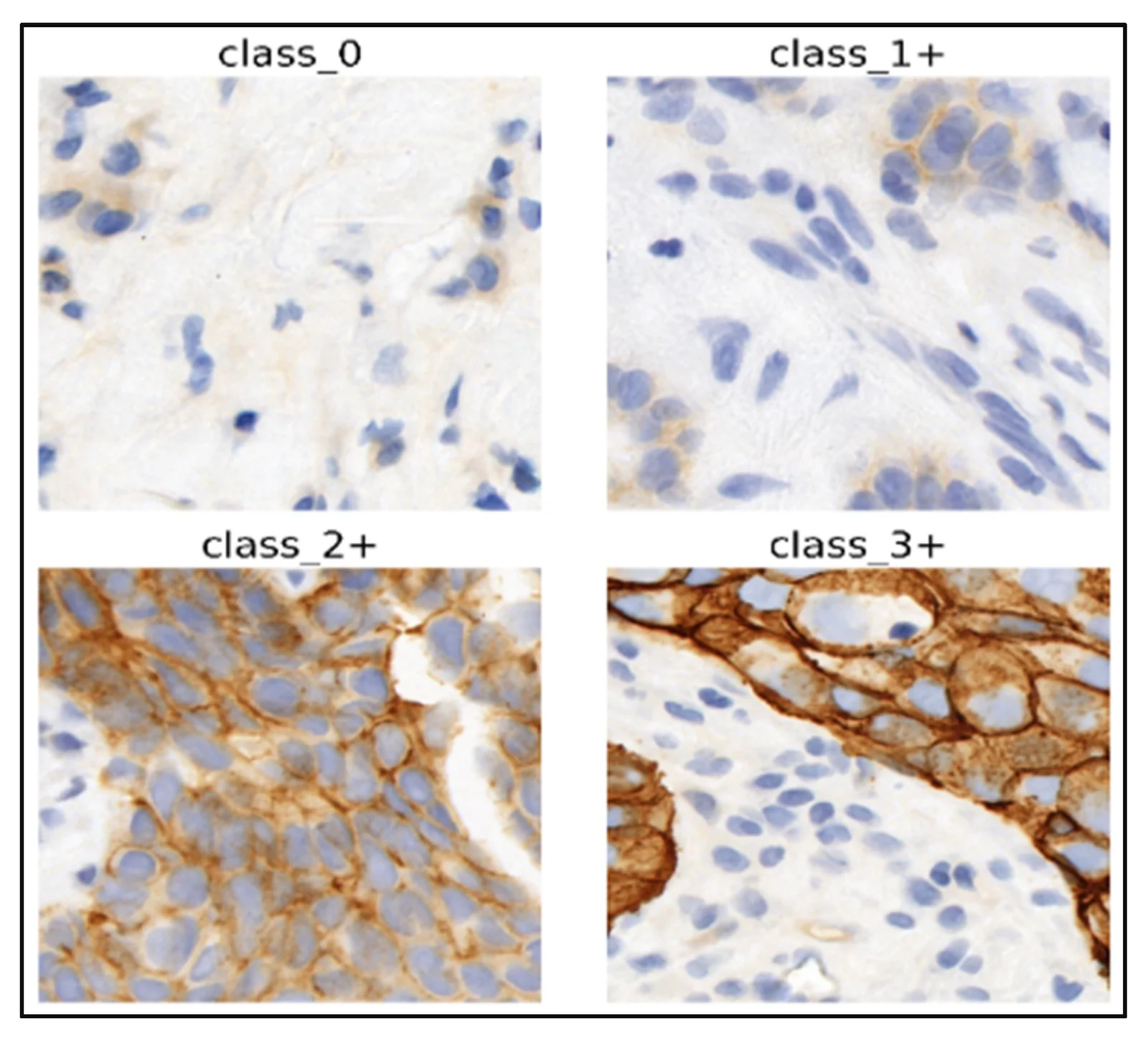
Representative samples from the HER2 public histopathology dataset, comprising HER2 classes 0, 1+, 2+, and 3+. The source whole-slide images were digitized at 40× magnification.

The HER2 histopathology dataset comprises 107 breast cancer whole slide images (WSIs) obtained from the University of Malaya Medical Center (UMMC) for human epidermal growth factor receptor 2 immunohistochemistry (IHC) grading. Digitized at 40× magnification with a 3DHistech Pannoramic DESK scanner (3DHISTECH Ltd., Budapest, Hungary), the WSIs underwent expert pathologist annotation to identify tumor regions of interest, from which 1024 × 1024 pixel patches were cropped. These image patches are categorized into four standard clinical expression scores—0 (negative), 1+ (low), 2+ (equivocal), and 3+ (positive)—as shown in Figure 2. For experimental evaluation, the dataset is partitioned into 8093 training patches and 1847 testing patches, with a 10% subset of the training data isolated for validation purposes [58].

(ii) NCT-CRC-HE-100K

**Figure 3:**
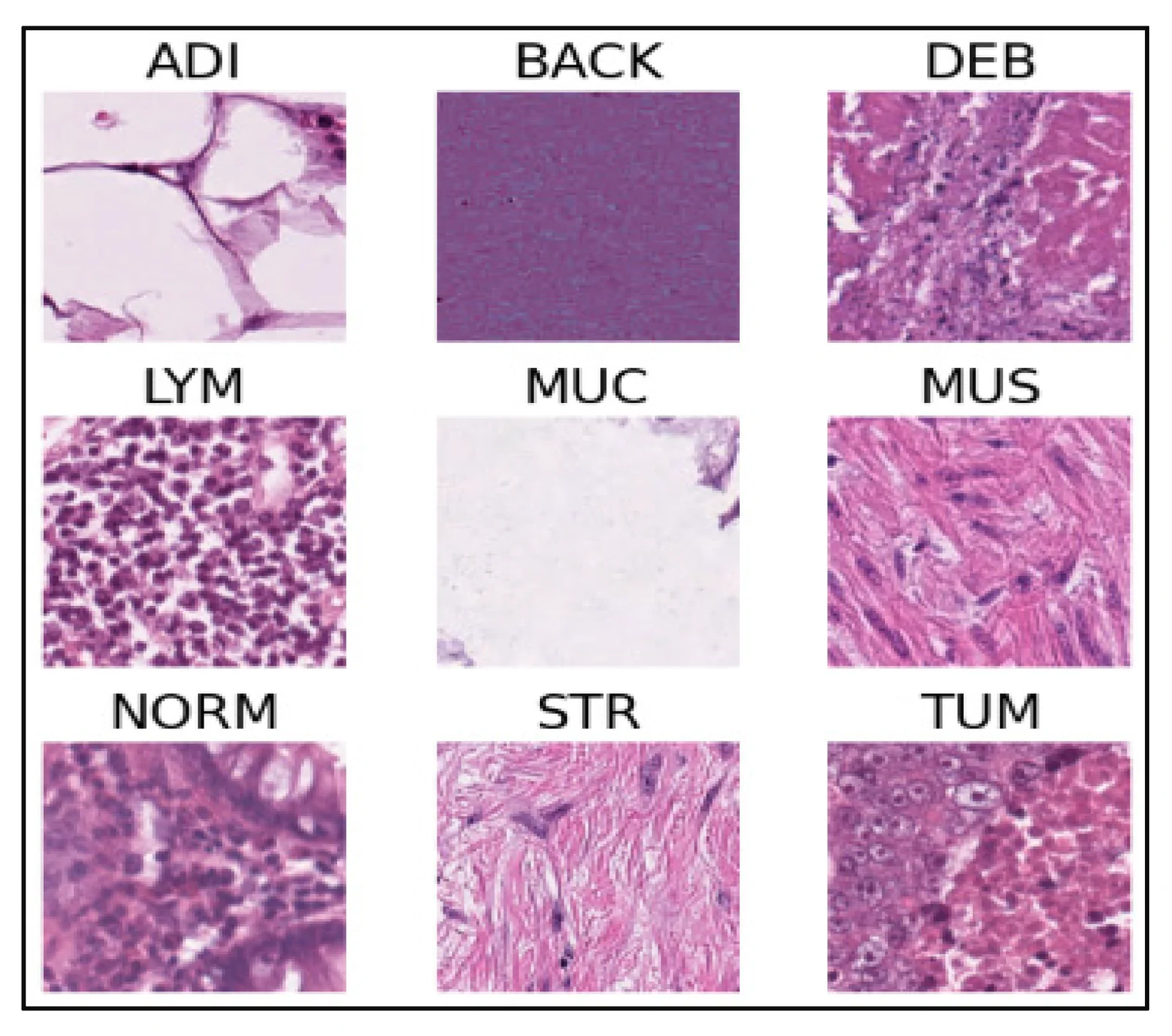
Representative samples from the NCT-CRC-HE-100K public histopathology dataset. The image patches were obtained at 20× magnification with a spatial resolution of 0.5 µm/pixel.

The NCT-CRC-HE-100K dataset [59] comprises roughly 100,000 hematoxylin and eosin (H&E) stained histopathology image patches derived from both colorectal cancer (CRC) and normal tissues. These non-overlapping patches were cropped from 86 whole-slide images sourced from the UMM Pathology Archive in Mannheim, Germany, and the National Center for Tumor Diseases in Heidelberg, Germany. Each digital tile measures 224 × 224 pixels, captured at 20× optical magnification with a spatial resolution of 0.5 μm per pixel. To ensure consistency across samples, the images underwent color normalization via the Macenko algorithm. The patches are distributed across nine distinct histological tissue classes: adipose tissue (ADI), background (BACK), cellular debris (DEB), lymphocytes (LYM), mucus (MUC), smooth muscle (MUS), normal colonic mucosa (NORM), cancer-associated stroma (STR), and colorectal adenocarcinoma epithelium (TUM), as shown in Figure 3.

(iii) CT Kidney

**Figure 4:**
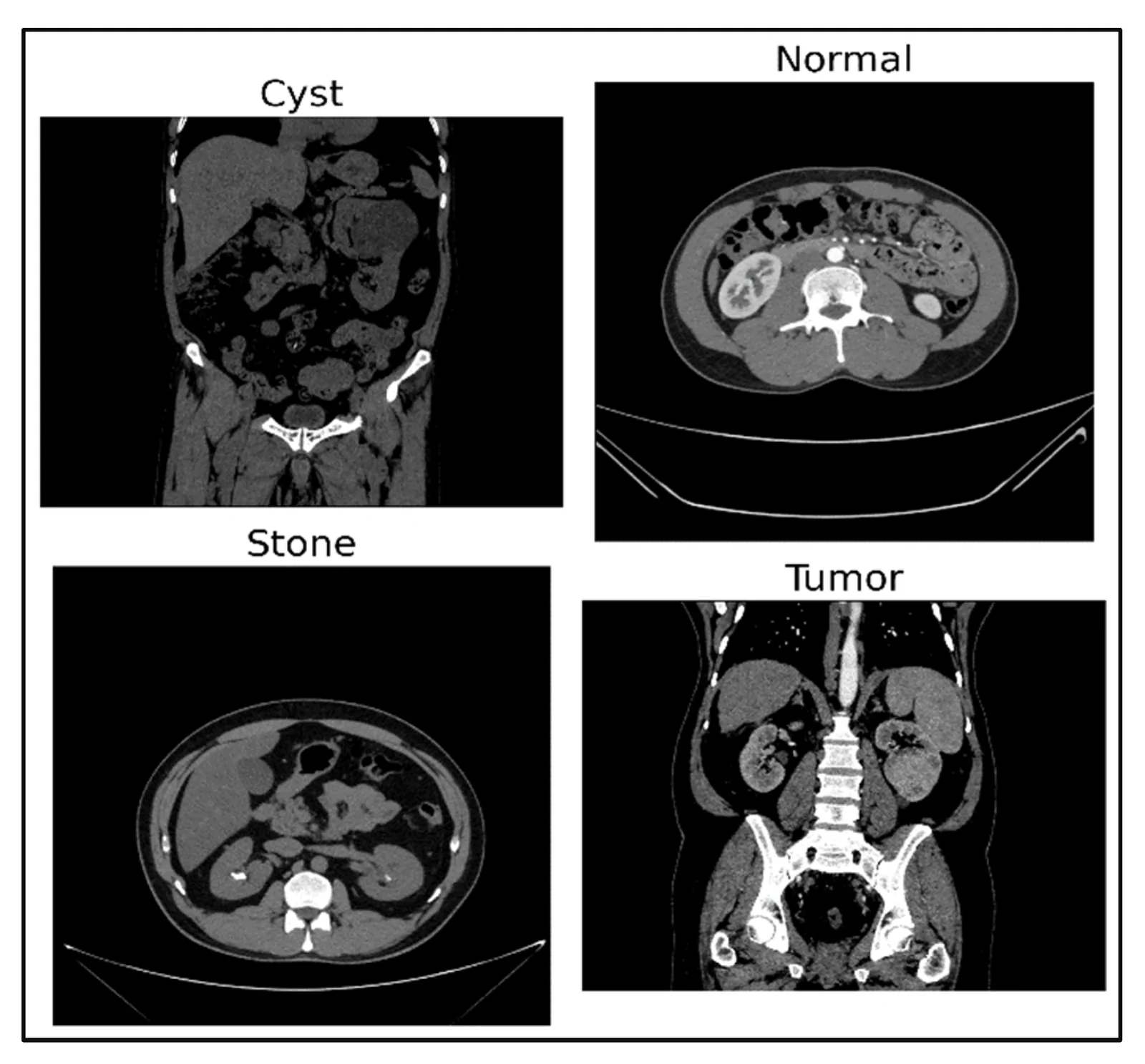
CT kidney public dataset sample.

The CT Kidney Dataset [60] comprises computed tomography images retrieved from the Picture Archiving and Communication Systems (PACS) of several medical institutions in Dhaka, Bangladesh. Covering a cohort of patients presenting with renal cysts, tumors, calculi, or healthy baselines, the repository aggregates 12,446 images partitioned into four clinical classes: Normal, Cyst, Tumor, and Stone, as shown in Figure 4. Specifically, the data distribution consists of 5077 normal, 3709 cyst, 2283 tumor, and 1377 stone instances. Every scan within the collection was systematically labeled and validated according to documented radiological findings.

(iv) HistoSet-5×14

**Figure 5:**
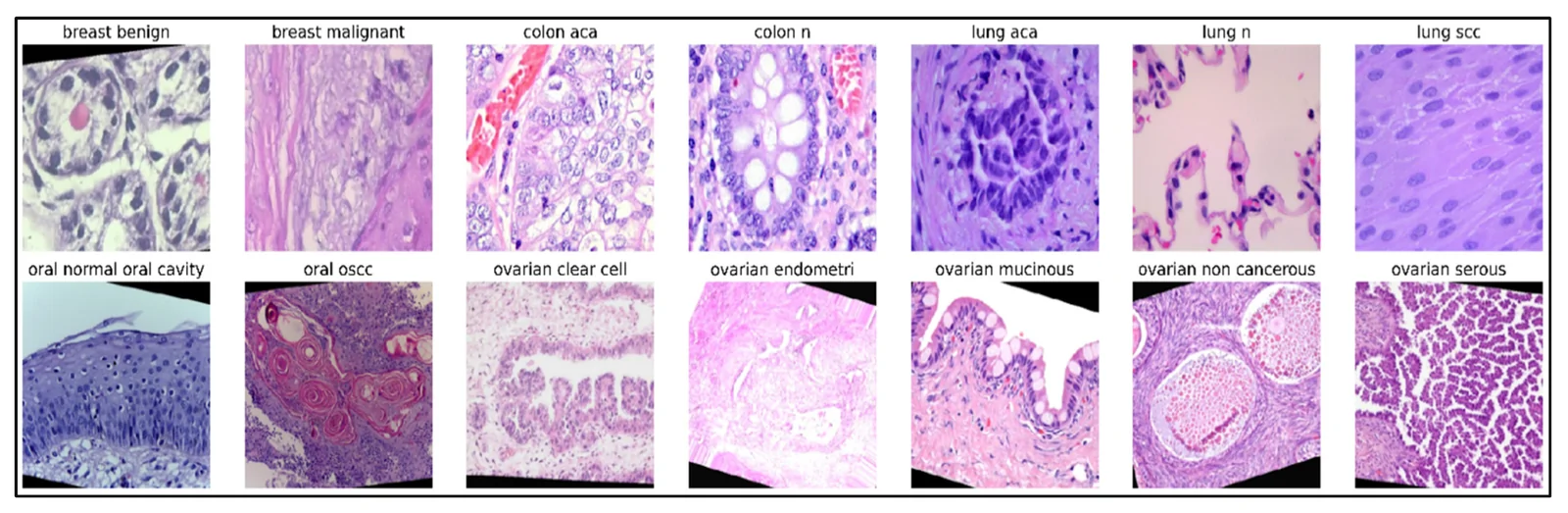
Representative samples from the HistoSet-5×14 public histopathology dataset. The dataset is assembled from multiple public histopathology sources with heterogeneous image-acquisition settings and magnifications; therefore, no single magnification applies to all samples.

The HistoSet-5×14 dataset is a standardized, curated repository developed to advance multi-class and multi-organ machine learning research in computational pathology by unifying hematoxylin and eosin (H&E)-stained tissue images from four public reference sources (LC25000, BreakHis, ORCHID, and an ovarian histopathology set). Spanning five organs—breast, colon, lung, oral cavity, and ovary (see Figure 5)—the collection encompasses fourteen distinct cancerous and non-cancerous tissue classes. To overcome severe class imbalances and sample heterogeneity of the original cohorts, a preprocessing pipeline combined strategic down-sampling with conservative data augmentation, preserving critical diagnostic morphology while balancing each category to exactly 2000 images for a total of 28,000 samples. The complete, standardized dataset is publicly accessible at https://www.kaggle.com/datasets/abidhasanrafi/histoset-5x14 (accessed on 15 January 2025).

### 3.2. Dataset Preprocessing

As evidenced by the distribution in Table 2, the raw training partition exhibits an extreme data skew, where the malignant majority class (NPC) outnumbers the ultra-minority class (LHP) by a ratio of nearly 78:1. Left unaddressed, this severe imbalance forces deep learning models to overfit to the majority features, leading to high overall accuracy but poor minority sensitivity. To construct a stable, robust training distribution without physically altering the dataset, a weighted random sampling strategy is employed during batch construction. For each class *c*, we compute its frequency *fc* in the training set. Sampling weights are then defined as the inverse of these frequencies in Equation (1):

(1) $$w_{c}=\frac{1}{f_{c}}$$

and normalized so that $\sum_{c}w_{c}=1$. For every training sample belonging to class *c*, an individual sampling weight equal to $w_{c}$ is assigned. The WeightedRandomSampler implemented in PyTorch version 2.10.0+cu128 was used to draw samples with replacement according to these per-sample weights. Consequently, minority classes receive a higher probability of being selected in each mini-batch, while the majority class is proportionally undersampled. The total number of samples drawn per epoch remains equal to the original dataset size, ensuring that the model sees a balanced class distribution stochastically across iterations.

Formally, let $D_{train}={\left\{(x_{i},y_{i}\right\}}_{i=1}^{N}$ be the original training set, with class labels $y_{i}\in \left\{1,\ldots ,K\right\}$ class frequencies $f_{c}=\left|\left\{i,y_{i}=c\right\}\right|$. For each sample *i*, we assign a sampling probability as shown in Equation (2).

(2) pi=1fyi∑j=1N1fyi

The WeightedRandomSampler generates a sequence of indices by drawing with replacement. The resulting mini-batches are formed from these indices, providing the network with an approximately uniform exposure to all classes throughout training. No explicit majority downsampling or minority augmentation injection is required; the sampler inherently balances the gradient updates while preserving all original samples.

For the controlled experiments reported in this study, all images were resized to 224 × 224 pixels, converted to RGB tensors, and normalized channel-wise using mean values [0.485, 0.456, 0.406] and standard deviations [0.229, 0.224, 0.225]. The same deterministic preprocessing procedure was applied to the training, validation, and test partitions. No stochastic augmentation, including random cropping, random resizing, horizontal or vertical flipping, rotation, or color transformations, was applied in these experiments. Class imbalance in the NPC training partition was addressed using the inverse-frequency WeightedRandomSampler described above rather than through image-level augmentation.

### 3.3. Proposed Model Architecture

The primary contribution of this work is the development of a lightweight yet effective Deep Convolutional Neural Network (DCNN) based on Depthwise Separable Convolutions and Residual Projection Learning. Unlike conventional VGG-style networks, which rely heavily on computationally expensive standard convolutions, the proposed architecture employs Depthwise Separable Convolutional Blocks (DSBlocks) to significantly reduce the number of trainable parameters and computational complexity while maintaining strong feature-extraction capability.

**Figure 6:**
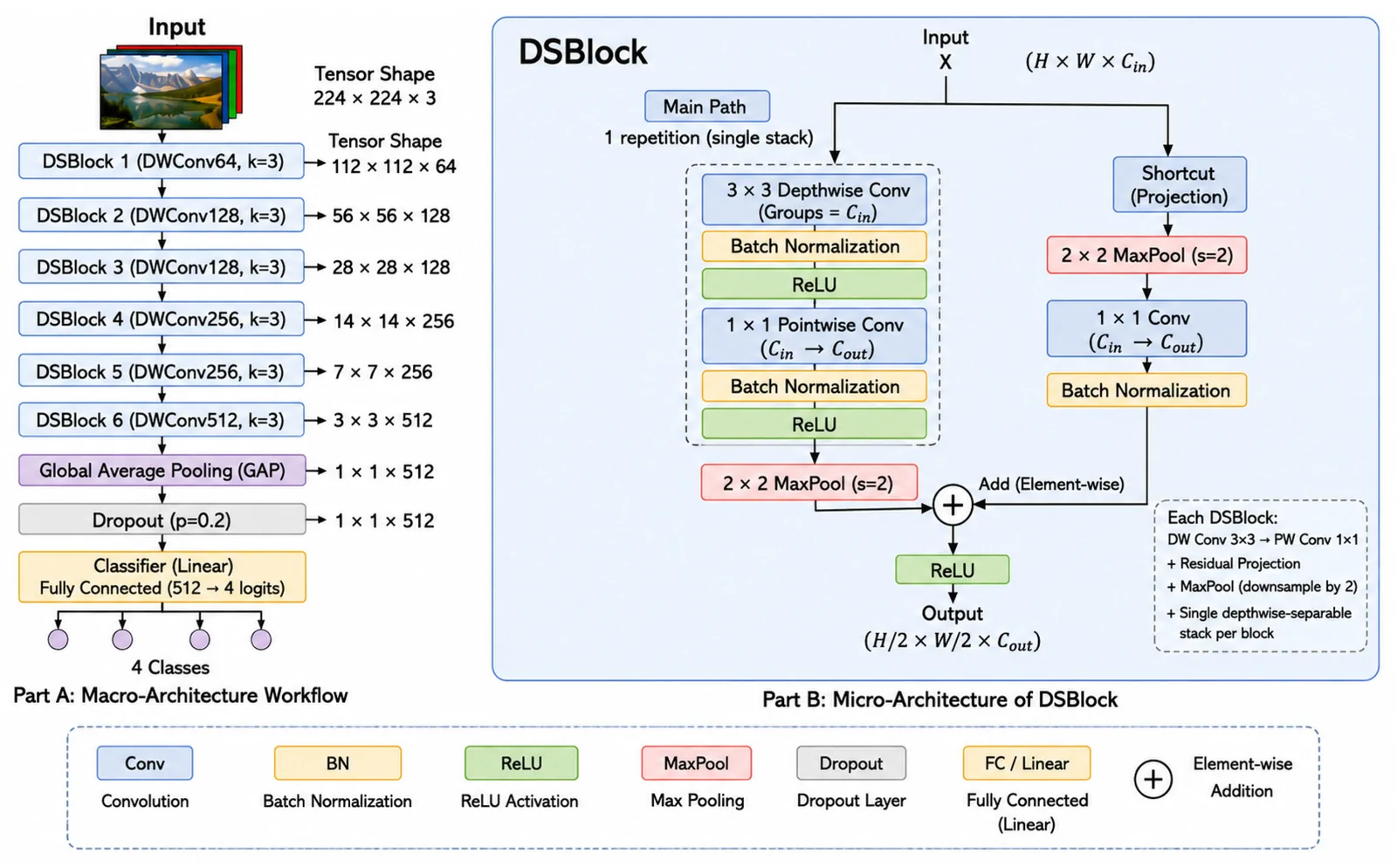
Proposed depthwise separable convolutional neural network architecture with residual projection. Part A illustrates the overall network workflow from the input image to the four-class classifier, while Part B presents the internal structure of the proposed DSBlock consisting of depthwise convolution, pointwise convolution, residual projection, batch normalization, ReLU activation, and max-pooling operations.

As illustrated in Figure 6, the proposed model consists of six sequential DSBlocks followed by a Global Average Pooling (GAP) layer, a dropout regularization layer, and a dropout layer (*p* = 0.2) followed by a fully connected linear classification layer producing four raw logits. The detailed network configuration, including the input/output dimensions and number of output filters for each stage, is summarized in Table 3.

#### 3.3.1. The Depthwise Separable Block (DSBlock)

The fundamental building block of the proposed architecture is the Depthwise Separable Block (DSBlock), whose internal structure is illustrated in Part B of Figure 6**.** Unlike standard convolutions that jointly learn spatial and cross-channel correlations, the DSBlock factorizes the convolution operation into two computationally efficient stages: a depthwise convolution followed by a pointwise convolution.

Given an input feature map X, each DSBlock applies a single depthwise convolution followed by batch normalization and ReLU activation, then a 1 × 1 pointwise convolution followed by batch normalization and ReLU. A 2 × 2 max-pooling operation reduces the spatial resolution before residual addition.

1. Depthwise Convolution: A spatial filter is applied independently to each input channel:

(3) $${\hat{G}}_{k,l,m}=\sum_{i,j}{\hat{K}}_{i,j,m}.X_{k+i-1,l+j-1,m}$$

where $\hat{K}$ represents the spatial kernel of size 3 × 3, and *m* indexes the current channel. This operation is immediately regularized via Batch Normalization (BN) and a Rectified Linear Unit (ReLU) activation function.

2. Pointwise Convolution: A 1 × 1 linear projection scales the channel depth to the target filter size $C_{out}:$

(4) $$G_{k,l,n}=\sum_{m=1}^{C_{in}}{\tilde{K}}_{1,m,n}.{\hat{G}}_{k,l,m}$$

where $\tilde{K}$ is the 1 × 1 pointwise kernel mapping $C_{in}⟶C_{out}$, followed by a secondary BN and ReLU activation.

#### 3.3.2. Linear Projection Residual Mapping

To preserve information flow across spatial transformations, we introduce a residual shortcut path within each block. Because the main processing path utilizes a Max-Pooling operation with a 2 × 2 window and a stride of 2 to reduce spatial dimensionality, the shortcut input must be adjusted to match these dimensions before element-wise addition.

The residual shortcut first applies 2 × 2 max-pooling to match the spatial downsampling of the main path, followed by a 1 × 1 projection convolution and batch normalization to match the target channel dimension. The projected shortcut is then added element-wise to the main-path output, followed by ReLU activation. The complete forward propagation through a single DSBlock is formalized in Equations (5)–(7):

X_main = MaxPool_2_ × _2_(F_DS(X)) (5)

X_shortcut = BN(Conv_1_ × _1_(MaxPool_2_ × _2_(X))) (6)

(7) $$Y_{block}=ReLU\left(X_{main}+X_{shorcut}\right)$$

where $F_{DS}$ denotes a single depthwise separable convolution stack comprising depthwise convolution, pointwise convolution, batch normalization, and ReLU activation. The residual projection ensures that both feature maps possess identical spatial dimensions and channel depths before element-wise addition.

#### 3.3.3. Macro-Architecture Layout

The overall architecture of the proposed model is illustrated in Figure 6, while its detailed layer-by-layer configuration is presented in Table 3**.** The network begins with an RGB input image of size (224 × 224 × 3) and processes it through six sequential DSBlocks. Each block progressively extracts higher-level feature representations while reducing the spatial resolution through max-pooling operations.

Unlike traditional CNN architectures that employ flattening operations followed by large fully connected layers, the proposed model utilizes GAP to substantially reduce parameter count and mitigate overfitting. The resulting 512-dimensional feature vector is regularized using a dropout layer with *p* = 0.2 and then passed to a final fully connected linear layer that produces four raw class logits. During training, these logits are supplied directly to the multi-class cross-entropy loss; an explicit softmax output layer is therefore not used in the model definition.

### 3.4. Model Training and Evaluation

The proposed DSR-VGG model was trained for a fixed 20 epochs using the AdamW optimizer with an initial learning rate of 3 × 10^−4^ and a weight-decay coefficient of 1 × 10^−4^. A mini-batch size of 32 was used throughout training. The learning rate was adjusted using the PyTorch CosineAnnealingLR scheduler with T_max = 20, corresponding to the complete 20-epoch training schedule. No validation-based early stopping or validation-selected checkpointing was applied; validation performance was monitored during training, and the final model after completion of the predefined 20 epochs was used for evaluation.

The network produces raw class logits at its final linear classification layer and is optimized using the multi-class Cross-Entropy Loss. For a mini-batch containing M samples and C diagnostic classes, the loss is defined as Equation (8):

(8) $$L=-\frac{1}{M}\sum_{i=1}^{M}\ln\left(\frac{\exp\left(z_{i,y_{i}}\right)}{\sum_{c=1}^{C}\exp\left(z_{i,c}\right)}\right)$$

where M is the mini-batch size, C is the number of diagnostic classes, z_i_,c denotes the raw logit produced for class c for the i-th sample, and y_i_ denotes its corresponding ground-truth class label. The softmax normalization required for cross-entropy computation is incorporated internally within the loss calculation rather than being applied as a separate output activation during training.

For the primary NPC dataset, severe class imbalance was addressed using the inverse-frequency WeightedRandomSampler described in Section 3.2. The sampler was applied only to the training partition, whereas the validation and test partitions retained their original class distributions. For the complete DSR-VGG model, training stability was additionally evaluated using three independent random seeds, namely 42, 13, and 2026. The controlled ablation experiments were performed using seed 42 while keeping all remaining training conditions unchanged, thereby allowing the effects of depthwise separable convolution, residual projection, and weighted sampling to be evaluated under a consistent experimental setting.

Table 4 presents the class-wise performance of DSR-VGG across three independent random seeds on the private NPC dataset, providing an assessment of training stability and variability. For the baseline comparison presented in Table 5, VGG16, ShuffleNet, MobileNet, EfficientNet, Swin Transformer, and ConvNeXt were initialized using ImageNet-pretrained weights and subsequently fine-tuned on the corresponding target datasets. To maintain closely matched experimental conditions, all baseline architectures used the same predefined dataset partitions, 224 × 224 input resolution, deterministic preprocessing procedure, mini-batch size of 32, training duration of 20 epochs, and initial learning rate of 3 × 10^−4^. The proposed DSR-VGG was trained from random initialization because pretrained weights are not available for the newly designed architecture. Accordingly, the comparison should be interpreted as an empirical evaluation under closely matched conditions rather than as evidence that all performance differences arise exclusively from architectural design.

Model performance was monitored using the validation partition during training, while the predefined patch-level test partition was reserved for final quantitative evaluation. Performance was derived from the multi-class confusion matrix using class-wise True Positives (TP), True Negatives (TN), False Positives (FP), and False Negatives (FN) in a one-vs-rest formulation. Multiclass accuracy was defined as the proportion of correctly classified samples among all *N* evaluated samples, where y_i_ and ŷ_i_ denote the true and predicted labels, respectively. The principal performance measures were Accuracy, Precision, Recall, and F1-score as shown in Equations (9)–(12):

(9) $$\mathrm{Accuracy}=(1/N){\sum_{i=1}}^{N}1(y_{i}={\hat{y}}_{i})$$

(10) $$Precision=\frac{TP}{TP+FP}$$

(11) $$Recall=\frac{TP}{TP+FN}$$

(12) $$F_{1-score}=2\times \frac{Precision\times Recall}{Precision+Recall}$$

Because the NPC dataset exhibits substantial class imbalance, macro averaging as shown in Equation (13) was used to summarize aggregate Precision, Recall, and F1-score. Each metric was first computed independently for each class using a one-vs-rest formulation, and the unweighted arithmetic mean was then calculated across all C classes. Consequently, each diagnostic category contributes equally to the reported macro metrics regardless of its number of test samples. Overall accuracy was calculated separately over all test samples.

(13) $$Macro\;Precision=\frac{1}{C}\sum_{c=1}^{C}Precision_{c}\;Macro\;Recall=\frac{1}{C}\sum_{c=1}^{C}Recall_{c}\;Macro\;F_{1}=\frac{1}{C}\sum_{c=1}^{C}F_{1,c}$$

To provide a concise summary of the complete DSR-VGG learning workflow, including weighted sampling, residual DSBlocks, model optimization, and evaluation, Algorithm 1 is presented below.
**Algorithm 1 DSR-VGG Training and Evaluation Procedure****Input:**Training, validation, and test patch-level partitions; image size 224 × 224; number of classes C; batch size B = 32; DSBlock channels [64, 128, 128, 256, 256, 512]; initial learning rate 3 × 10^−4^; weight decay 1 × 10^−4^; dropout *p* = 0.2; epochs E = 20; T_max = 20; random seed.**Output:**Final DSR-VGG model and test results: accuracy, balanced accuracy, class-wise precision/recall/F1, macro-precision, macro-recall, macro-F1, weighted F1, confusion matrix, parameter count, FLOPs, and single-image inference latency.**Step****Procedure****1**Set the random seed and load the predefined training, validation, and test patch-level partitions.**2**Resize each image to 224 × 224, convert to an RGB tensor, and normalize channel-wise using mean [0.485, 0.456, 0.406] and standard deviation [0.229, 0.224, 0.225]. Apply the same deterministic preprocessing to all partitions; no stochastic image augmentation is used.**3**For the NPC training partition, compute inverse-frequency sample weights and construct a WeightedRandomSampler with replacement and num_samples equal to the training-set size. Preserve the original validation and test class distributions.**4**Initialize DSR-VGG with six sequential DSBlocks. In each block, apply the main path DW Conv 3 × 3 → BN → ReLU → PW Conv 1 × 1 → BN → ReLU → MaxPool 2 × 2. Apply the shortcut MaxPool 2 × 2 → Conv 1 × 1 → BN, add the two paths element-wise, and apply ReLU.**5**Apply global average pooling, Dropout (*p* = 0.2), and a Linear layer (512 → C) to produce raw class logits.**6**Initialize multi-class CrossEntropyLoss, AdamW with learning rate 3 × 10^−4^ and weight decay 1 × 10^−4^, and CosineAnnealingLR with T_max = 20.**7**For each of the 20 epochs, train on weighted mini-batches: perform the forward pass, compute cross-entropy loss, back-propagate gradients, and update parameters using AdamW.**8**At the end of each epoch, evaluate the validation partition for monitoring and update the cosine-annealing learning-rate scheduler. No validation-based early stopping is applied.**9**After completion of the predefined 20 epochs, evaluate the final model once on the predefined test partition. Obtain predicted labels using argmax over the raw logits.**10**Compute the confusion matrix, overall accuracy, balanced accuracy, class-wise precision/recall/F1, macro-precision, macro-recall, macro-F1, and weighted F1.**11**For stability analysis, repeat the complete DSR-VGG training with seeds 42, 13, and 2026. Conduct the controlled ablation experiments with seed 42 under otherwise identical settings.**12**Profile parameter count, FLOPs, and single-image inference latency. Report public-dataset experiments separately as supplementary architectural-robustness evidence rather than external clinical validation of NPC diagnosis.

## 4. Results and Discussion

### 4.1. NPC Classification Performance of DSR-VGG

Table 4 summarizes the class-wise classification performance of the complete DSR-VGG model across three independent training runs using random seeds 42, 13, and 2026. Performance is reported as the mean ± standard deviation to characterize the stability of the model under different random initializations. Across the three runs, DSR-VGG achieved an overall classification accuracy of 99.764 ± 0.049%, a balanced accuracy of 99.070 ± 0.365%, a macro-averaged precision of 99.373 ± 0.089%, a macro-averaged recall of 99.070 ± 0.365%, and a macro-F1 score of 99.219 ± 0.215%. These results demonstrate consistently high classification performance across the four diagnostic categories despite the substantial class imbalance in the NPC dataset.

The NPC and NPI classes exhibited particularly stable performance across the three runs. NPC achieved a mean precision of 99.892 ± 0.012%, recall of 99.851 ± 0.055%, and F1-score of 99.872 ± 0.031%, while NPI achieved 99.332 ± 0.229% precision, 99.753 ± 0.035% recall, and 99.542 ± 0.133% F1-score. The small standard deviations indicate that classification performance for these two classes was highly consistent across the independent training runs.

The Normal class also demonstrated stable recognition performance, with a mean precision of 99.352 ± 0.263%, recall of 99.264 ± 0.091%, and F1-score of 99.308 ± 0.134%. The minority LHP class exhibited slightly greater variation, which is expected given its substantially smaller number of available samples. Nevertheless, LHP maintained a mean precision of 98.916 ± 0.281%, recall of 97.412 ± 1.395%, and F1-score of 98.156 ± 0.842% across the three runs. These results indicate that strong minority-class recognition was maintained across independent model initializations, although the larger standard deviation in LHP recall reflects the greater uncertainty associated with its limited sample support.

The clinical relevance of distinguishing morphologically related tissue patterns is further illustrated by the broader spectrum of sinonasal pathology. For example, sinonasal inverted papilloma may coexist with inflammatory changes, squamous metaplasia, and squamous cell carcinoma, illustrating that benign, inflammatory, metaplastic, and malignant changes can occur within closely related anatomical settings [61]. Such pathological heterogeneity reinforces the importance of reliable differentiation among the Normal, LHP, NPI, and NPC categories considered in the present study. Overall, DSR-VGG achieved an accuracy of 99.764 ± 0.049% across three independent runs, demonstrating strong internal patch-level discrimination among the four diagnostic categories.

The clinical importance of reliable NPC recognition is further emphasized by the variable presentation of the disease. NPC may occasionally present with atypical neurological manifestations, and histopathological biopsy may be required for definitive diagnosis when initial clinical or radiological findings are inconclusive [62]. In the present study, the NPC class achieved a mean precision of 99.892 ± 0.012%, recall of 99.851 ± 0.055%, and F1-score of 99.872 ± 0.031% across three independent runs. Although these results demonstrate strong internal patch-level discrimination, confirmation using patient-/WSI-disjoint and independent clinical cohorts remains necessary before the model can be considered for diagnostic-support applications.

The very high patch-level performance should also be interpreted in the context of the dataset and evaluation protocol. The images were annotated by three expert pathologists using Cytomine 2025 platform, although the available documentation does not specify a formal consensus procedure or interobserver-agreement statistic. Acquisition-specific visual regularities may also remain across the two contributing institutions. Most importantly, because the original split was performed at the patch level, correlated patches originating from the same WSI may occur across the training, validation, and test subsets. These factors may contribute to optimistic internal performance estimates. The results should therefore be interpreted as strong internal patch-level discrimination rather than evidence of patient-level, WSI-level, or external clinical generalization.

**Figure 7:**
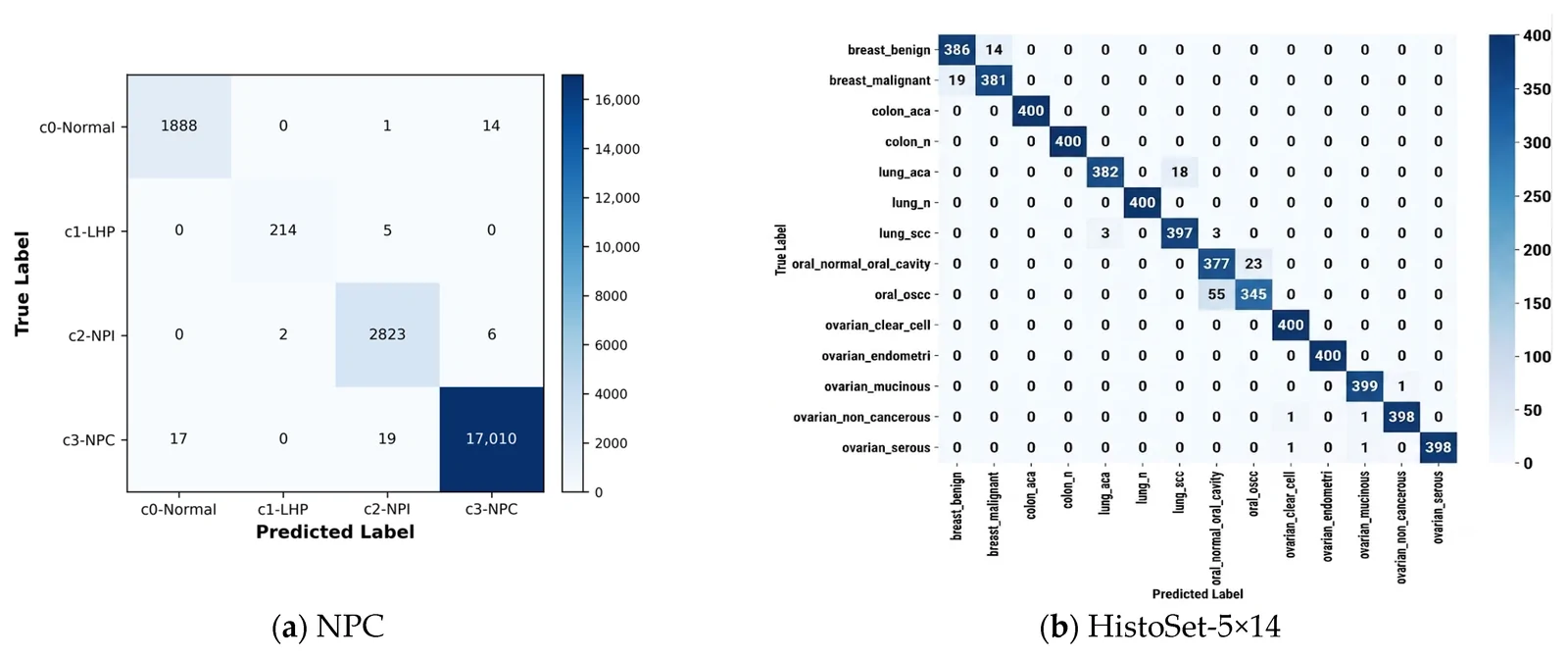
Confusion matrices of the proposed DSR-VGG model for the NPC and HistoSet-5×14 datasets. (**a**) Representative seed-42 confusion matrix for the NPC test set, with rows indicating true classes and columns indicating predicted classes; (**b**) confusion matrix for the HistoSet-5×14 dataset. Both matrices illustrate the class-wise prediction performance of the proposed model.

The confusion matrix in Figure 7a corresponds to the representative frozen seed-42 model rather than the three-seed average. For this run, DSR-VGG correctly classified 21,935 of 21,999 test patches, corresponding to an accuracy of 99.709%. Specifically, 1888 of 1903 Normal patches, 214 of 219 LHP patches, 2823 of 2831 NPI patches, and 17,010 of 17,046 NPC patches were correctly classified. The seed-42 LHP recall was therefore 97.717%, with five LHP samples misclassified as NPI. This representative confusion matrix is consistent with the strong class-wise performance observed across the three independent runs.

### 4.2. Comparison with Existing Baseline Models

Table 5 presents a comparison between DSR-VGG and several widely adopted deep learning architectures, including VGG16, ShuffleNet, MobileNet, EfficientNet, Swin Transformer, and ConvNeXt, on the private NPC dataset and the multi-histopathology HistoSet-5×14 dataset. In Table 5, the results obtained by DSR-VGG are highlighted in bold to distinguish the proposed architecture from the baseline models. The comparison shows that DSR-VGG achieves a favorable trade-off between predictive performance and model complexity, rather than absolute superiority in classification accuracy across all models.On the private NPC dataset, the proposed model achieved an accuracy of 99.709%. Several baseline architectures achieved marginally higher raw classification accuracy, including EfficientNet, ShuffleNet, Swin Transformer, and MobileNet. EfficientNet achieved the highest accuracy of 99.93%, while ShuffleNet, Swin Transformer, and MobileNet achieved 99.84%, 99.86%, and 99.78%, respectively. However, the four-class DSR-VGG model contains only approximately 0.528 million trainable parameters, compared with 1.27 million for ShuffleNet, 2.24 million for MobileNet, 4.03 million for EfficientNet, 27.53 million for Swin Transformer, 27.83 million for ConvNeXt, and 134.3 million for VGG16. Therefore, the principal advantage of DSR-VGG lies in preserving highly competitive predictive performance with a substantially smaller parameter footprint rather than achieving the highest absolute classification accuracy.

A similar pattern is observed on the HistoSet-5×14 dataset. The proposed model achieved an overall accuracy of 98.00%, while ConvNeXt achieved the highest accuracy of 99.54%. Nevertheless, the proposed architecture remained competitive while retaining a substantially more compact design than the larger baseline networks. These results support the use of DSR-VGG as a parameter-efficient architecture capable of maintaining strong predictive performance without relying on excessive network capacity. The confusion matrices shown in Figure 7a,b further illustrate the class-wise prediction patterns of the proposed architecture on the NPC and HistoSet-5×14 datasets.

**Figure 8:**
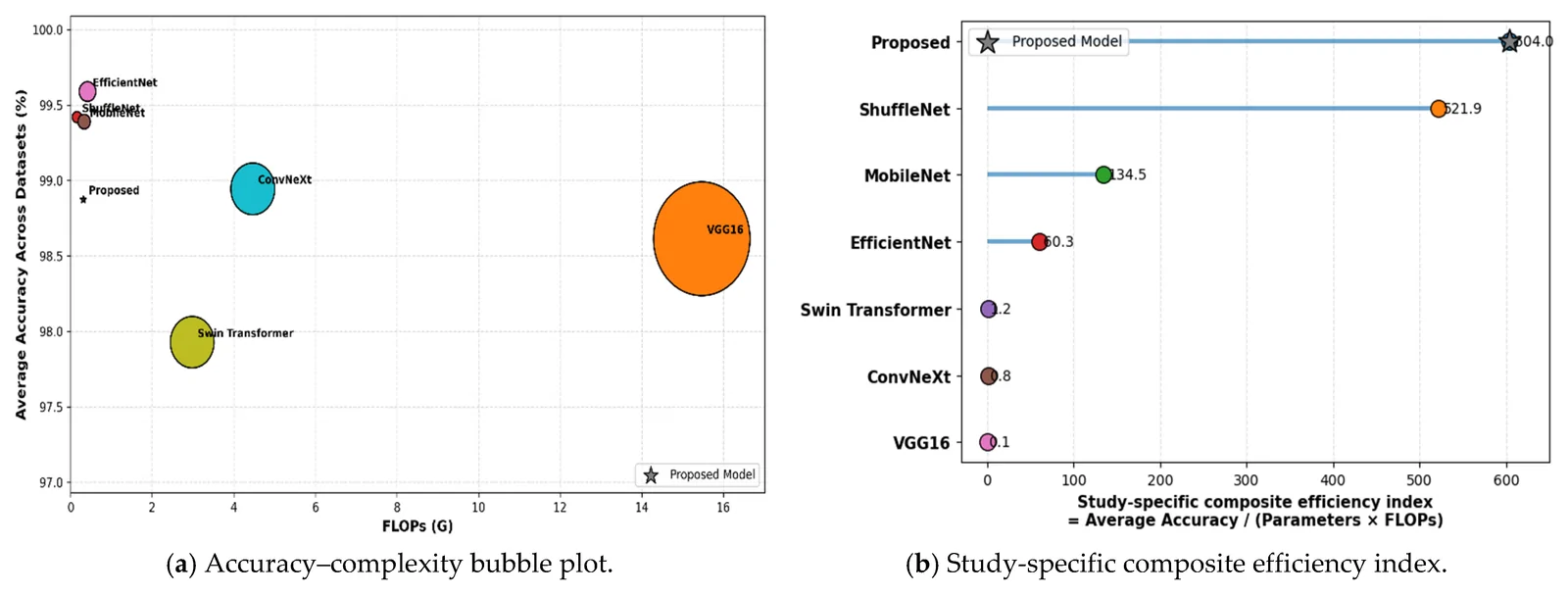
Computational trade-off visualization: (**a**) average classification accuracy versus FLOPs, with bubble size representing parameter count; (**b**) study-specific composite efficiency index.

Beyond predictive performance, computational complexity is an important consideration when evaluating lightweight deep learning models. As illustrated in Figure 8a, DSR-VGG has the smallest parameter footprint among the evaluated architectures. For the four-class NPC configuration, its approximately 0.528 million parameters correspond to reductions of more than 99% relative to heavyweight architectures such as VGG16, Swin Transformer, and ConvNeXt. Compared with lightweight baselines, DSR-VGG also uses fewer parameters than ShuffleNet, MobileNet, and EfficientNet. However, parameter count and arithmetic workload describe different aspects of computational efficiency. For example, although DSR-VGG has a smaller parameter count than ShuffleNet, ShuffleNet has a lower reported FLOP count. Computational efficiency should therefore be interpreted using multiple complementary measures rather than a single indicator.

To provide a supplementary visualization of the trade-off between classification performance and model complexity, a study-specific composite efficiency index is defined in Equation (14) as classification accuracy divided by the product of parameter count and FLOPs. For this within-study comparison, accuracy is expressed in percentage (%), parameter count in millions (M), and computational complexity in GFLOPs (G). Figure 8b uses the average accuracy across the NPC and HistoSet-5×14 comparisons. The index is intended only as a descriptive within-study measure; it is not a standardized or universal measure of model efficiency, and its numerical magnitude depends on the selected units and multiplicative formulation. It also does not account for memory-access patterns, peak memory use, hardware architecture, implementation efficiency, energy consumption, or other system-level factors. Accordingly, the individual accuracy, parameter count, FLOPs, and latency values reported in Table 6 remain the primary basis for computational comparison.

Figure 8b therefore should be interpreted as illustrating the relative positioning of the evaluated architecture in terms of predictive performance and computational complexity rather than demonstrating universal superiority of any single model. Although several baseline models achieved slightly higher classification accuracy, DSR-VGG maintained competitive predictive performance with a markedly smaller parameter footprint. The results indicate that the main contribution of the proposed architecture is its parameter-efficient accuracy–complexity trade-off, supported by the integration of depthwise separable convolution and residual learning.

The supplementary evaluation on heterogeneous public datasets further indicates that the proposed architecture can maintain competitive performance across datasets with different image characteristics. These experiments are interpreted as evidence of architectural robustness and transferability, rather than as evidence of clinical generalization across diseases or institutions. Together with its compact model size, the results suggest that DSR-VGG is a promising candidate for further evaluation in resource-constrained computing environments. Nevertheless, patient-/WSI-disjoint validation, institution-wise evaluation, prospective testing, and complete WSI-level workflow assessment remain necessary before conclusions regarding clinical deployment can be established.

(14) $$Efficiency=\frac{Accuracy}{Parameters\times FLOPs}$$

### 4.3. Computational Complexity Analysis

To evaluate the computational characteristics of the proposed model, a benchmarking analysis was conducted against six established deep learning architectures. As summarized in Table 6, the models were compared in terms of parameter count, FLOPs, and inference latency. DSR-VGG contains only approximately 0.528 million trainable parameters, making it approximately 254× smaller than VGG16, 2.4× smaller than ShuffleNet, 4.2× smaller than MobileNet, and 7.6× smaller than EfficientNet. It is also substantially more compact than Swin Transformer and ConvNeXt, which contain 27.53 million and 27.83 million parameters, respectively. These results demonstrate that DSR-VGG achieves a highly compact architectural design while maintaining competitive classification performance.

Inference-latency benchmarking was conducted in a Kaggle computational environment equipped with two NVIDIA Tesla T4 GPUs (T4 × 2) (NVIDIA Corporation, Santa Clara, CA, USA). All evaluated architectures were benchmarked under the same hardware and software environment using single-image inference with a batch size of 1. Under these conditions, DSR-VGG achieved the lowest measured latency of 2.38 ms per image, compared with 5.41 ms for MobileNet, 6.81 ms for ShuffleNet, 8.88 ms for EfficientNet, 9.53 ms for VGG16, 12.72 ms for Swin Transformer, and 6.24 ms for ConvNeXt. Although ShuffleNet has a lower theoretical computational workload of 0.15 GFLOPs compared with 0.31 GFLOPs for DSR-VGG, DSR-VGG exhibited lower measured inference latency within the same benchmarking environment. This observation illustrates that theoretical computational complexity and practical inference speed do not necessarily vary proportionally.

Overall, the combination of a 0.528-million-parameter footprint, low computational complexity, and 2.38 ms single-patch inference latency demonstrates the computational efficiency of DSR-VGG and supports its potential for high-throughput histopathological patch analysis and resource-constrained deployment. Because inference latency is hardware- and implementation-dependent, the reported values should be interpreted specifically within the stated Kaggle benchmarking environment and are intended primarily for relative comparison among the evaluated architectures. Furthermore, the reported latency represents patch-level model inference only and does not include image acquisition, WSI tiling, preprocessing, data transfer, or other components of an end-to-end clinical workflow. Evaluation of complete WSI processing and end-to-end deployment latency therefore remains an important direction for future work.

### 4.4. Ablation Study

To quantify the individual contributions of the principal components of DSR-VGG, a controlled ablation study was conducted by separately examining depthwise separable convolution, residual projection, and weighted random sampling. Four configurations were evaluated under identical data partitions and training settings: (1) the complete DSR-VGG incorporating depthwise separable convolution, residual projection, and weighted sampling; (2) a variant without residual projection; (3) a residual variant in which the depthwise-pointwise convolutions were replaced by standard 3 × 3 convolutions; and (4) the complete DSR-VGG trained without weighted sampling. The results are summarized in Table 7, where bold values indicate the highest performance obtained for each reported metric.

The complete DSR-VGG achieved an accuracy of 99.709%, balanced accuracy of 99.109%, and macro-F1 of 99.201%. Removing the residual projection reduced accuracy to 99.332%, balanced accuracy to 97.570%, and macro-F1 to 97.804%. The reduction was more evident in the class-balanced metrics than in overall accuracy, indicating that residual projection contributes to stable feature propagation and balanced recognition across the four diagnostic classes.

The standard-convolution residual variant achieved slightly higher accuracy of 99.768% and macro-F1 of 99.457% than the depthwise-separable DSR-VGG. Thus, the present controlled experiment does not indicate that depthwise separable convolution improves predictive performance. Its principal advantage is computational efficiency. The standard-convolution variant required approximately 2.552 million parameters, whereas DSR-VGG required only 0.528 million, representing an approximately 79.3% reduction in parameter count. Similarly, the convolution/linear computational workload decreased from approximately 1.930 GMAC to 0.274 GMAC, corresponding to an approximately 85.8% reduction. Therefore, depthwise separable convolution enables DSR-VGG to retain near-equivalent classification performance with substantially lower computational complexity.

Weighted sampling showed a different contribution. Removing the WeightedRandomSampler resulted in a marginally higher overall accuracy of 99.723%, compared with 99.709% when weighted sampling was employed. However, balanced accuracy decreased from 99.109% to 98.007%, and macro-F1 decreased from 99.201% to 98.539%. The effect was particularly evident for the severely underrepresented LHP class: recall decreased from 97.717% to 93.607%, while F1-score decreased from 98.391% to 95.571% when weighted sampling was removed. These findings indicate that weighted sampling primarily improves minority-class recognition and class-balanced performance rather than headline overall accuracy.

Overall, the ablation study demonstrates distinct roles for the three components. Residual projection supports balanced predictive performance, weighted sampling improves recognition of the minority LHP class, and depthwise separable convolution provides the principal computational-efficiency advantage while maintaining competitive classification performance.

### 4.5. Supplementary Cross-Dataset Robustness Evaluation

This subsection presents supplementary evaluations of DSR-VGG on publicly available medical image-classification datasets, including CT Kidney Stone, HER2, and CRC-100K. These experiments are intended to assess the cross-dataset robustness and computational efficiency of the proposed architecture under different medical image-classification settings. Because these datasets differ from the primary NPC dataset in disease site, tissue type, staining characteristics, class definitions, and, in the case of CT Kidney Stone, imaging modality, the results should not be interpreted as external clinical validation of NPC diagnosis.

As shown in Table 8, DSR-VGG achieved competitive performance across the CT Kidney Stone, HER2, and CRC-100K datasets while requiring substantially lower computational resources than many compared approaches. On the CT Kidney Stone dataset, DSR-VGG attained 99.96% accuracy, precision, recall, and F1-score. This exceeds the 99.20% accuracy reported by Mahmood et al. [63] while using approximately 0.528 M parameters and 0.31 GFLOPs. These results provide supplementary evidence that the compact DSR-VGG architecture can learn discriminative representations across heterogeneous medical image-classification settings.

**Figure 9:**
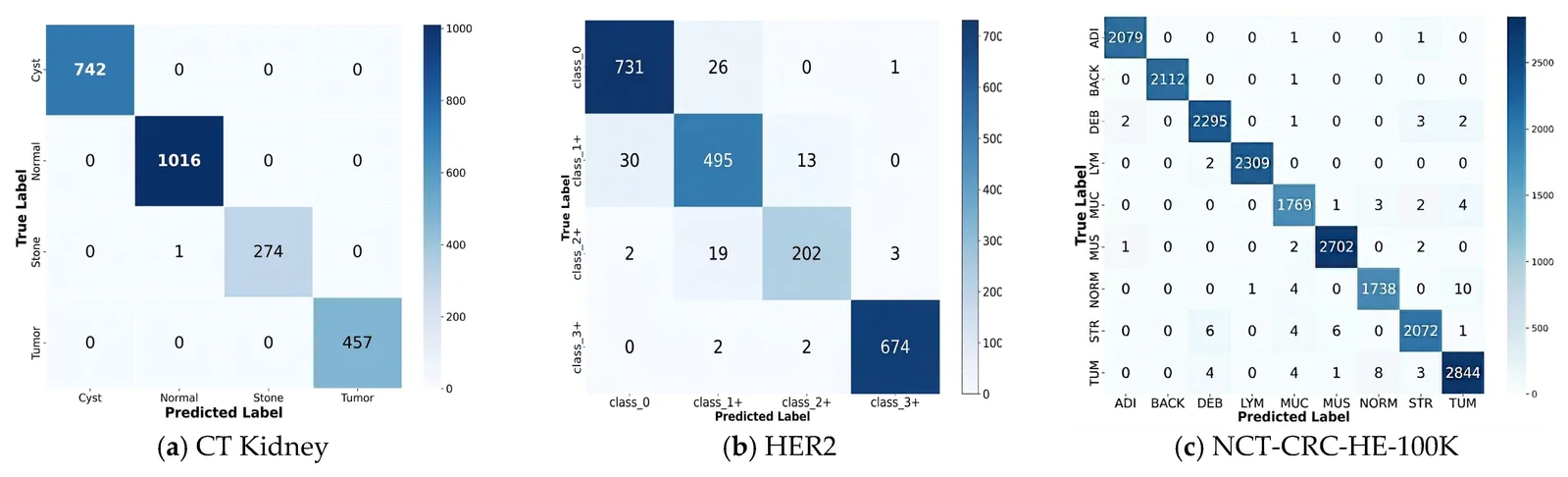
Confusion matrices of the proposed DSBlock-based model for the CT Kidney, HER2, and NCT-CRC-HE-100K public benchmark datasets.

On the HER2 dataset, the proposed method achieved 95.55% accuracy, marginally below Hasan et al. [68] at 95.61%, while reducing computational cost from 2.10 GFLOPs to 0.31 GFLOPs and using approximately 0.528 M parameters compared with 0.972 M for Hasan et al. [68]. On the CRC-100K dataset, DSR-VGG achieved 99.60% accuracy and F1-score, slightly below Pacal and Attallah [69] at 99.96%, while using approximately 0.531 M parameters compared with 26.38 M. It also maintained a compact computational profile relative to Khalid et al. [70] and Hasan et al. [68]. Figure 9a–c show strong class-wise prediction patterns across the three supplementary benchmark datasets. Overall, these experiments provide evidence of architectural robustness and transferability across heterogeneous medical image-classification tasks rather than external clinical generalization of the NPC classifier. Independent patient-/WSI-disjoint multi-center validation, prospective testing, and complete WSI-level evaluation remain necessary before clinical applicability can be established.

### 4.6. Statistical Robustness and Qualitative Explainability

To quantify the statistical uncertainty of the representative seed-42 evaluation, 5000 class-stratified nonparametric bootstrap resamples were generated from the held-out test predictions. The resulting patch-level accuracy was 99.709% (95% percentile CI: 99.636–99.777%), while macro-F1 was 99.201% (95% CI: 98.844–99.501%). For the minority LHP class $\left(n=219\right),$ recall was 97.717% (95% CI: 95.434–99.543%) and F1-score was 98.391% (95% CI: 97.025–99.541%). These confidence intervals quantify uncertainty at the patch level and should not be interpreted as independent-patient confidence intervals because the bootstrap procedure does not account for correlations among patches originating from the same patient or WSI. Training stability across three independent random seeds (42, 13, and 2026) is reported separately in Table 4, where the mean ± standard deviation is provided for the overall and class-wise performance metrics. The patch-level bootstrap confidence intervals for the representative seed-42 model are summarized in Table 9.

**Figure 10:**
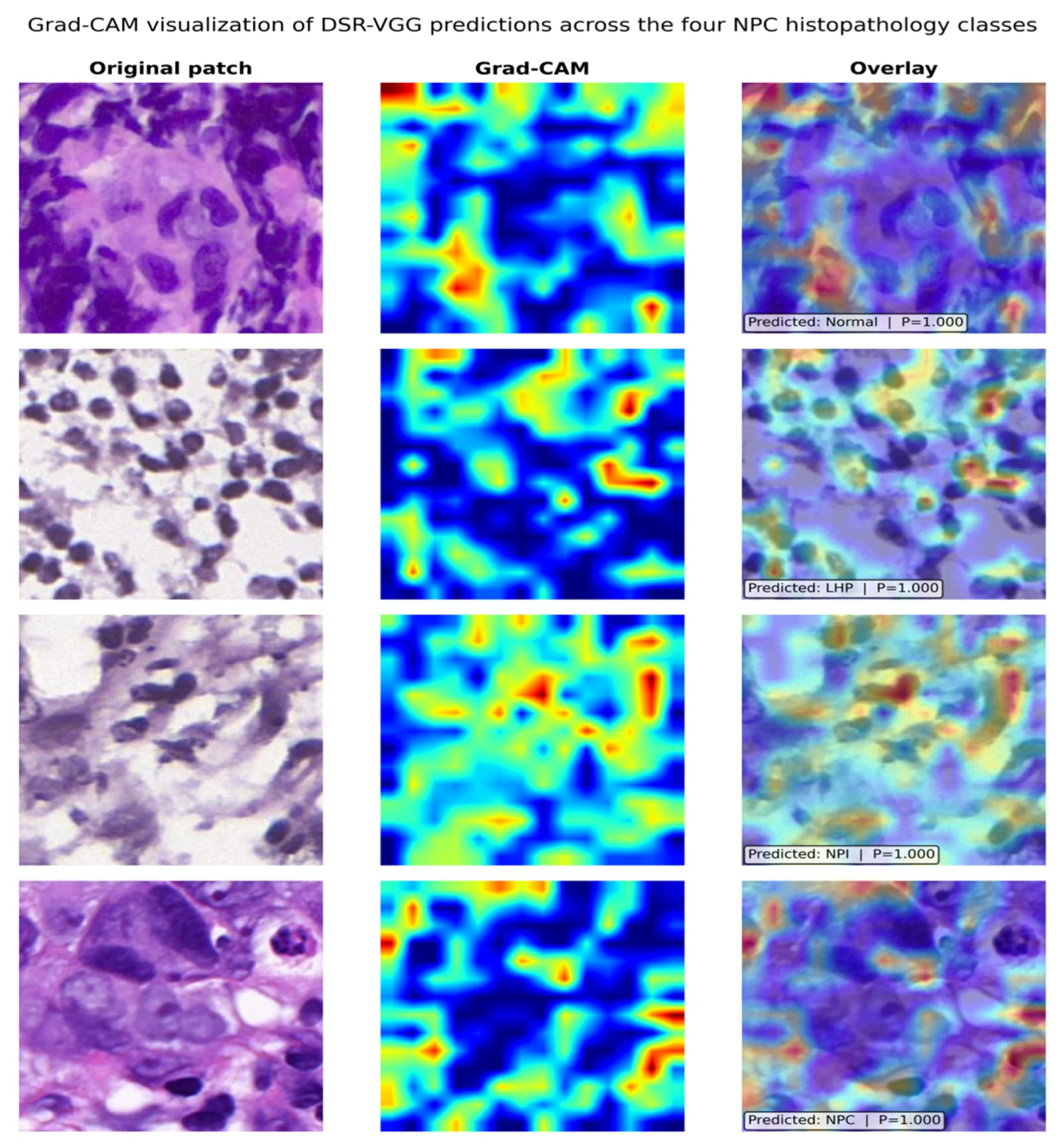
Grad-CAM visualizations of representative correctly classified Normal, LHP, NPI, and NPC histopathological patches using the pointwise convolution of the fifth DSBlock. Each row presents the original image, the interpolated Grad-CAM activation map, and the corresponding overlay. Warmer colors indicate regions contributing more strongly to the predicted class score. The source whole-slide images from Sarawak General Hospital (SGH) were digitized at 20× magnification, whereas those from Hospital Kuala Lumpur (HKL) were digitized at 40× magnification. The activation maps are qualitative indicators of model attention and have not been independently validated as specific histopathological structures by a pathologist.

Grad-CAM was generated without retraining from the frozen seed-42 full-model checkpoint. Representative correctly predicted samples were chosen by a reproducible sharpness rule within a fixed, randomly selected pool of three correct samples per class. The pointwise convolution in DSBlock 5 provided 14 × 14 native feature maps prior to interpolation (the corresponding final-block map was 7 × 7). The overlays are qualitative indicators of model-attended regions, not pathologist-verified causal or morphological explanations. Representative Grad-CAM visualizations for the four NPC dataset classes are presented in Figure 10.

### 4.7. Limitations and Future Validation

A key limitation of the present study is that the original NPC dataset was randomly partitioned at the patch level rather than at the patient or WSI level. Consequently, correlated patches originating from the same WSI may occur across the training, validation, and test subsets, introducing a potential risk of information leakage and optimistic estimation of patch-level classification performance. The results should therefore be interpreted as internal patch-level performance rather than evidence of patient-level, WSI-level, or prospective clinical generalization. In addition, although the NPC dataset contains samples acquired from SGH and HKL under different imaging magnifications, verified center-to-patch mapping was not available during the present revision to support a reliable institution-wise evaluation. Future studies should therefore employ explicitly patient-/WSI-disjoint partitioning, assess cross-institutional generalization through center-wise training and testing, and include independent external clinical cohorts. The additional experiments conducted on heterogeneous public benchmark datasets provide complementary evidence of architectural robustness and transferability across different image domains; however, they do not substitute for patient-/WSI-disjoint or institution-wise validation on the primary NPC cohort.

## 5. Conclusions

This study presented DSR-VGG, a lightweight Depthwise Separable Residual VGG architecture for nasopharyngeal histopathological image classification. Inspired by VGG-style hierarchical feature extraction, the proposed architecture replaces computationally expensive standard convolutions with depthwise-pointwise separable convolutions and employs a simplified configuration consisting of a single DSBlock per stage. Residual projection shortcuts are incorporated to support information propagation and optimization while maintaining a compact network design. An inverse-frequency-weighted random sampling strategy is additionally employed to improve recognition of underrepresented classes in the highly imbalanced NPC dataset.

Experimental results demonstrate that DSR-VGG provides a favorable trade-off between predictive performance and computational efficiency. Across three independent runs, the model achieved an overall accuracy of 99.764 ± 0.049%, balanced accuracy of 99.070 ± 0.365%, and macro-F1 score of 99.219 ± 0.215% on the NPC dataset. The architecture contains only 0.528 million parameters, requires approximately 0.31 GFLOPs, and achieved a measured single-image inference latency of 2.38 ms under the stated GPU benchmarking conditions. Controlled ablation experiments showed that residual connections improved balanced classification performance, while weighted random sampling enhanced minority-class recognition, particularly for LHP. The standard-convolution variant achieved slightly higher predictive performance but at substantially greater parameter and computational cost, confirming that the principal benefit of depthwise separable convolution is improved computational efficiency rather than absolute classification superiority. Comparative evaluation against VGG16, MobileNet, ShuffleNet, EfficientNet, Swin Transformer, and ConvNeXt further showed that DSR-VGG maintains highly competitive predictive performance with a substantially smaller computational footprint.

Supplementary experiments on heterogeneous public medical image-classification datasets further support the architectural robustness and transferability of the proposed efficiency-oriented design. Overall, the findings indicate that DSR-VGG offers a compact and computationally efficient framework for histopathological patch classification. Future work should extend the proposed approach toward independent multi-center validation, patient-/WSI-disjoint evaluation, and whole-slide inference to further assess its applicability in pathology workflows.

## Figures and Tables

**Table 1:** Summary of datasets used in the study.

Dataset	Role	Modality/Stain	Classes	Total Samples	Data Split Used (%) Training/Val/Test	Remarks
NPC Private	Primary dataset	WSI histopathology	4	88,002 patches	50/25/25	SGH and HKL; 20× and 40×
HER2-IHC-40×	Supplementary	Breast IHC histopathology	4	10,997	80/-/20	HER2 scores 0, 1+, 2+, 3+
NCT-CRC-HE-100K	Supplementary	Colorectal H&E histopathology	9	99,959	80/-/20	224 × 224 patches
HistoSet-5×14	Supplementary	Multi-organ H&E histopathology	14	28,000	80/-/20	2000 images per class
CT Kidney	Supplementary cross-dataset benchmark	CT images	4	12,446	80/-/20	Different modality from pathology

Note. WSI = whole-slide image; IHC = immunohistochemistry; H&E = hematoxylin and eosin; SGH = Sarawak General Hospital; HKL = Hospital Kuala Lumpur.

**Table 2:** Numerical distribution and class split of the nasopharyngeal dataset.

Class	Training	Validation	Test
NPC	34,093	17,047	17,046
NPI	5665	2832	2831
LHP	439	220	219
NORMAL	3805	1902	1903
Total	44,002	22,001	21,999
%Distribution	50%	25%	25%

**Table 3:** Architecture details of the proposed model.

Layer/Block	Component Layers	Input Resolution	Output Resolution	Output Filters
Input	Raw Image Tensor	224 × 224 × 3	224 × 224 × 3	3
DSBlock 1	Main path: DW Conv 3 × 3 → BN → ReLU → PW Conv 1 × 1 (64) → BN → ReLU → MaxPool 2 × 2. Shortcut: MaxPool 2 × 2 → 1 × 1 projection Conv (64) → BN → residual addition → ReLU	224 × 224 × 3	112 × 112 × 64	64
DSBlock 2	Main path: DW Conv 3 × 3 → BN → ReLU → PW Conv 1 × 1 (128) → BN → ReLU → MaxPool 2 × 2. Shortcut: MaxPool 2 × 2 → 1 × 1 projection Conv (128) → BN → residual addition → ReLU	112 × 112 × 64	56 × 56 × 128	128
DSBlock 3	Main path: DW Conv 3 × 3 → BN → ReLU → PW Conv 1 × 1 (128) → BN → ReLU → MaxPool 2 × 2. Shortcut: MaxPool 2 × 2 → 1 × 1 projection Conv (128) → BN → residual addition → ReLU	56 × 56 × 128	28 × 28 × 128	128
DSBlock 4	Main path: DW Conv 3 × 3 → BN → ReLU → PW Conv 1 × 1 (256) → BN → ReLU → MaxPool 2 × 2. Shortcut: MaxPool 2 × 2 → 1 × 1 projection Conv (256) → BN → residual addition → ReLU	28 × 28 × 128	14 × 14 × 256	256
DSBlock 5	Main path: DW Conv 3 × 3 → BN → ReLU → PW Conv 1 × 1 (256) → BN → ReLU → MaxPool 2 × 2. Shortcut: MaxPool 2 × 2 → 1 × 1 projection Conv (256) → BN → residual addition → ReLU	14 × 14 × 256	7 × 7 × 256	256
DSBlock 6	Main path: DW Conv 3 × 3 → BN → ReLU → PW Conv 1 × 1 (512) → BN → ReLU → MaxPool 2 × 2. Shortcut: MaxPool 2 × 2 → 1 × 1 projection Conv (512) → BN → residual addition → ReLU	7 × 7 × 256	3 × 3 × 512	512
GAP	Global Average Pooling 2D	3 × 3 × 512	1 × 1 × 512	512
Dropout	*p* = 0.2	512	512	512
Classifier	Linear output layer (raw logits)	512	4	4

**Table 4:** Class-wise classification performance of DSR-VGG across three independent random seeds. Values are reported as mean ± standard deviation (%).

Class/Overall Metric	Precision (%)	Recall (%)	F1-Score (%)
Normal	99.352 ± 0.263	99.264 ± 0.091	99.308 ± 0.134
LHP	98.916 ± 0.281	97.412 ± 1.395	98.156 ± 0.842
NPI	99.332 ± 0.229	99.753 ± 0.035	99.542 ± 0.133
NPC	99.892 ± 0.012	99.851 ± 0.055	99.872 ± 0.031
Macro Average	99.373 ± 0.089	99.070 ± 0.365	99.219 ± 0.215
Overall Accuracy	99.764 ± 0.049	—	—
Balanced Accuracy	99.070 ± 0.365	—	—

**Table 5:** Performance comparison of DSR-VGG and baseline deep learning architectures on the NPC private and HistoSet-5×14 datasets. Precision, Recall, and F1-score are reported using macro averaging.

Dataset	Model	Acc (%)	Macro Precision (%)	Macro Recall (%)	Macro F1-Score (%)
NPC Private	VGG16	99.62	98.67	98.59	98.63
	ShuffleNet	99.84	99.62	99.38	99.50
	MobileNet	99.78	99.30	99.31	99.31
	EfficientNet	99.93	99.80	99.67	99.73
	Swin Transformer	99.86	98.96	99.12	99.04
	ConvNeXt	98.35	93.50	96.48	94.95
	**DSR-VGG**	**99.71**	**99.30**	**99.11**	**99.20**
HistoSet-5 × 14	VGG16	97.61	97.61	97.61	97.60
	ShuffleNet	99.00	99.00	99.00	99.00
	MobileNet	99.00	99.00	99.00	99.00
	EfficientNet	99.25	99.20	99.30	99.20
	Swin Transformer	96.00	96.00	96.00	95.00
	ConvNeXt	99.54	99.54	99.54	99.54
	**DSR-VGG**	**98.00**	**98.00**	**98.00**	**98.00**

**Table 6:** Comparison of model complexity and inference efficiency in terms of parameters, relative model size, FLOPs, and latency across the proposed method and baseline architectures.

Model	Param. (M)	FLOPs (G)	Latency (ms)	Relative Size vs. Proposed
**DSR-VGG**	**0.528**	**0.31**	**2.38**	**1×**
VGG16	134.30	15.47	9.53	254.4× larger
ShuffleNet	1.27	0.15	6.81	2.4× larger
MobileNet	2.24	0.33	5.41	4.2× larger
EfficientNet	4.03	0.41	8.88	7.6× larger
Swin Transformer	27.53	2.98	12.72	52.1× larger
ConvNeXt	27.83	4.46	6.24	52.7× larger

**Table 7:** Controlled ablation analysis of the principal components of DSR-VGG.

Configuration	DWS	Residual	Weighted	Params (M)	Accuracy (%)	Balanced Acc. (%)	Macro-F1 (%)	LHP Recall (%)	LHP F1 (%)
Full **DSR-VGG**	✓	✓	✓	0.528	99.709	99.109	99.201	**97.717**	**98.391**
No residual	✓	✗	✓	0.270	99.332	97.570	97.804	—	—
Standard Conv + residual	✗	✓	✓	2.552	**99.768**	**99.320**	**99.457**	—	—
No weighted sampling	✓	✓	✗	0.528	99.723	98.007	98.539	93.607	95.571

Note: ✓ indicates that the corresponding component is included, whereas ✗ indicates that the corresponding component is excluded. Bold values indicate the highest value in each performance metric column.

**Table 8:** Comparison of the proposed model with state-of-the-art methods on the CT Kidney Stone, HER2, and CRC-100K datasets in terms of classification performance and computational complexity.

Dataset	Study	Acc (%)	Pre (%)	Recall (%)	F1 (%)	Param (M)	FLOPs (G)
CT Kidney Stone	Khan [64]	91.31	89.25	88.25	88.50	N/A	N/A
	Sharon and Anbarasi [65]	98.80	97.50	96.75	96.75	2.07	N/A
	Zhang et al. [66]	98.14	86.66	96.33	92.51	N/A	N/A
	Mahmood et al. [63]	99.20	99.10	99.30	99.50	N/A	N/A
	**DSR-VGG**	**99.96**	**99.96**	**99.96**	**99.96**	**0.528**	**0.31**
HER2	Nabi et al. [67]	93.00	93.00	93.00	92.00	N/A	N/A
	Hasan et al. [68]	95.61	92.76	95.63	94.04	0.972	2.10
	**DSR-VGG**	**95.55**	**95.54**	**95.55**	**95.54**	0.528	**0.31**
CRC-100K	Hasan et al. [68]	96.23	95.88	95.18	95.21	0.972	2.10
	Pacal and Attallah [69]	99.96	99.96	99.96	99.96	26.38	N/A
	Khalid et al. [70]	98.96	99.37	98.80	98.64	1.82	0.22
	Zhong et al. [71]	95.50	N/A	N/A	N/A	N/A	N/A
	**DSR-VGG**	**99.60**	**99.60**	**99.60**	**99.60**	0.531	**0.31**

Note: Bold values indicate the results obtained by the proposed DSR-VGG model, distinguishing them from the results of the other state-of-the-art methods.

**Table 9:** Patch-level bootstrap 95% confidence intervals for the representative seed-42 DSR-VGG model on the NPC test partition.

Metric	Seed-42 Point Estimate	95% Patch-Level CI
Accuracy	99.709%	99.636–99.777%
Balanced accuracy	99.109%	98.563–99.561%
Macro-F1	99.201%	98.844–99.501%
Weighted-F1	99.709%	99.636–99.777%
LHP Recall	97.717%	95.434–99.543%
LHP F1-score	98.391%	97.025–99.541%

## Data Availability

The primary dataset used in this study consists of private histopathological images obtained through a collaborative research agreement with Hospital Kuala Lumpur (HKL) and Sarawak General Hospital (SGH). Owing to patient confidentiality, ethical considerations, and institutional data-sharing restrictions, these data are not publicly available. Access to the private dataset may be considered upon reasonable request, subject to approval by the respective institutions and relevant ethics committees. The public benchmark datasets used for comparative evaluation are publicly available from their respective repositories, as cited in the manuscript.
